# Butyrate Deficiency due to Microbial Dysbiosis Promotes Anxiety

**DOI:** 10.34133/research.1470

**Published:** 2026-11-09

**Authors:** Lianyu Zhou, Zhenle Zang, Hang Zhang, Xinghang Fei, Gaohui Zhu, Yuan Mu, Peiyan Jiang, Xingyu Wang, Min Zhao, Zhaoyi Ai, Xiaoyuan Cao, Zhi Liu, Huachun Yin, Li Li, Jingjing Tian, Hui Yang, Xiaotang Fan, Shuai Tan, Song Li

**Affiliations:** ^1^ Department of Neurosurgery, Xinqiao Hospital, Army Medical University, Chongqing, China.; ^2^ Chongqing Institute for Brain and Intelligence, Guangyang Bay Laboratory, Chongqing, China.; ^3^ Yu-Yue Pathology Scientific Research Center, Jinfeng Laboratory, Chongqing, China.; ^4^ Department of Pathology, Southwest Hospital, Army Medical University, Chongqing, China.; ^5^ Department of Endocrinology, National Clinical Research Center for Child Health and Disorders, Children’s Hospital of Chongqing Medical University, Chongqing Medical University, Chongqing, China.; ^6^ School of Basic Medical Sciences, Chengdu University of Traditional Chinese Medicine, Chengdu, Sichuan, China.; ^7^ Department of Military Cognitive Psychology, School of Psychology, Army Medical University, Chongqing, China.

## Abstract

Individuals with diabetes frequently suffer from anxiety; however, the mechanism for this association remains incompletely understood. Diabetes-associated genes have been implicated in the control of central nervous system (CNS) disorders. In the current study, we observed a marked down-regulation of secretagogin (SCGN) across multiple organs in diabetic mouse models, and *Scgn* knockout (*Scgn*^−/−^) mice developed diabetes, confirming SCGN as a diabetes-associated gene. Behavioral analyses revealed that *Scgn*^−/−^ mice exhibited anxiety-like phenotypes compared to wild-type (WT) mice. Notably, germ-free (GF) *Scgn*^−/−^ mice did not manifest these behavioral abnormalities, suggesting that SCGN influences anxiety through gut microbiota-dependent mechanisms. Subsequent shotgun metagenomic sequencing confirmed gut microbiota dysbiosis in *Scgn*^−/−^ mice. Fecal microbiota transplantation (FMT) from *Scgn*^−/−^ donors into GF mice recapitulated the anxiety-like phenotypes. Given the established roles of microbial metabolites in gut–brain axis signaling, we identified a substantial depletion of butyrate, a key neuroactive metabolite, in fecal and plasma samples from *Scgn*^−/−^ mice. Immunohistochemical analysis revealed neural overactivation, as reflected by c-Fos expression in the medial prefrontal cortex (mPFC) of *Scgn*^−/−^ mice, which was corroborated by metabolomic profiling indicating dysregulated tryptophan metabolism in this region. Mechanistically, up-regulation of indoleamine 2,3-dioxygenase 1 (IDO1) and kynureninase (KYNU) led to excessive production of quinolinic acid, an NMDA receptor agonist that increased pyramidal neuron excitability in the mPFC, thereby promoting anxiety-like behaviors in *Scgn*^−/−^ mice. Supplementation with *Odoribacter splanchnicus,* a butyrate-producing bacterium, could reverse this abnormal signaling pathway and alleviate anxiety-like behaviors. Critically, butyrate administration mitigated this anxiogenic cascade in vivo and in vitro, as validated by electrophysiological recordings. Taken together, our findings demonstrate that SCGN deficiency induces gut dysbiosis, reduces butyrate availability, and promotes excitotoxicity in the mPFC, at least in part, through disrupted tryptophan metabolism, ultimately leading to anxiety. This SCGN-modulated gut–brain axis highlights potential new therapeutic targets for the treatment of diabetes-associated anxiety.

## Introduction

Diabetes is a prevalent chronic metabolic disease worldwide, posing marked threats to both physical health and psychological well-being [1]. Clinical and epidemiological studies have shown that anxiety disorder prevalence is substantially higher in diabetes patients compared to the general population [2,3]. However, the mechanisms underlying the comorbidity of diabetes and anxiety remain incompletely elucidated. Emerging evidence indicates that dysregulated glucose metabolism may induce anxiety through disruptions in neurotransmitter balance, compromised neuronal energy supply, and altered hypothalamic–pituitary–adrenal (HPA) axis function [4,5]. Accumulating evidence also demonstrates that diabetes-related genetic factors contribute substantially to the pathogenesis of anxiety disorders. Multiple diabetes susceptibility genes exhibit high expression within the central nervous system (CNS), where they influence neuronal activity, neurotransmitter release, and synaptic plasticity, consequently disrupting key neural circuits governing emotional regulation and elevating anxiety risk [2,6,7]. Notably, specific diabetes-related genes, such as *KCNJ11* and *IRS1*, appear to modulate functional integrity in anxiety-relevant brain regions such as the medial prefrontal cortex (mPFC) and amygdala. For instance, mutations in *KCNJ11*, which encodes the Kir6.2 subunit of pancreatic adenosine triphosphate-sensitive potassium (KATP) channels, are associated with neurodevelopmental disorders like autism spectrum disorder (ASD) and/or attention deficit hyperactivity disorder (ADHD) alongside anxiety disorders in patients with neonatal diabetes [8]. Similarly, IRS1, a critical component of insulin signaling, regulates cerebral glucose metabolism and neurotransmission dynamics. Dysregulation of IRS1 signaling has been shown to contribute to both insulin resistance and behaviors indicative of anxiety. Studies using animal models reveal that normalized IRS1 phosphorylation ameliorates lipopolysaccharide-induced insulin signaling deficits while concurrently mitigating anxiety/depressive-like behaviors [9]. These collective findings indicate that diabetes-related genes not only regulate peripheral glucose homeostasis but also critically shape emotional and anxiety responses through central neural circuitry.

In addition, the gut microbiota functions as an important internal environmental regulator, potentially influencing the development of diabetes-related anxiety [10–12]. Substantial evidence indicates altered gut microbiota composition and metabolic profiles in anxiety disorder patients, implicating that microbial dysbiosis may contribute to the underlying disease mechanisms [13,14]. Probiotic interventions and studies on fecal microbiota transplantation (FMT) further support the notion that the gut microbiota can modulate emotional states through the gut–brain axis [15–17]. Diabetes-related genetic factors, neural circuit abnormalities, and gut microbial dysbiosis act synergistically to facilitate the onset of anxiety disorders in diabetic individuals [18,19]. Elucidating these interconnected mechanisms is crucial for optimizing therapeutic strategies and disease management.

Secretagogin (SCGN) is a calcium-binding protein that is widely expressed in multiple organs, including the pancreas, gastrointestinal tract, thyroid, adrenal medulla, and CNS. It fundamentally regulates intracellular calcium signaling and associated physiological functions [20]. Extensive research has linked SCGN to a range of pathologies, such as diabetes mellitus, autism, autosomal recessive early-onset ulcerative colitis, and neurodegenerative disorders [21–24]. Additionally, clinical epidemiology consistently shows a higher prevalence of anxiety disorders among patients with diabetes and ulcerative colitis, which complicates disease management and substantially impairs quality of life through underlying pathophysiological mechanisms that are still not fully understood [3,25]. Our previous investigations have established that SCGN dysregulation precipitates autism-like phenotypes via aberrant proinflammatory responses and hormonal secretion imbalances [26]. However, whether and how SCGN affects the comorbidity of diabetes and anxiety remains unknown.

In the present study, we employed integrated molecular and biochemical approaches to evaluate anxiety-like behaviors in a diabetic model lacking SCGN systematically. To delineate the pathophysiological contributions of diabetes to anxiety, we dissected the molecular alterations within anxiety-regulating neural circuits while characterizing the metabolic profiles of the gut microbiota. We observed that diabetic *Scgn* knockout (*Scgn*^−/−^) mice exhibited pronounced anxiety-like phenotypes. Mechanistically, diabetes induced gut microbiota dysbiosis with a consequent considerable reduction in butyrate production. This metabolic impairment disrupted tryptophan metabolism within the mPFC and subsequently elevated quinolinic acid (QA) levels, which led to hyperexcitability in mPFC pyramidal neurons and ultimately promoted anxiety behaviors. These findings establish a foundation for the development of new therapeutic strategies for the clinical management of anxiety comorbidity with diabetes.

## Results

### Diabetes-related gene SCGN mediates anxiety-like behaviors via the microbiota

Our investigation employed the well-established *db/db* diabetic mouse model, exhibiting obesity (Fig. S1A) and significantly elevated blood glucose levels (Fig. S1B). Initial analysis revealed reduced SCGN protein expression across multiple organs in *db/db* mice, including the brain, thyroid, adrenal gland, intestine, and pancreas (Fig. 1A to C), whereas the expression of SCGN was undetectable in the liver, consistent with prior reports [27]. Subsequent analyses of *Scgn*^−/−^ mice demonstrated substantially impaired glucose metabolism during the intraperitoneal glucose tolerance test (IPGTT) (Fig. S1C and D), recapitulating the diabetic phenotype observed in *db/db* mice. These findings suggest that SCGN deficiency is associated with diabetes.

**Fig. 1.**
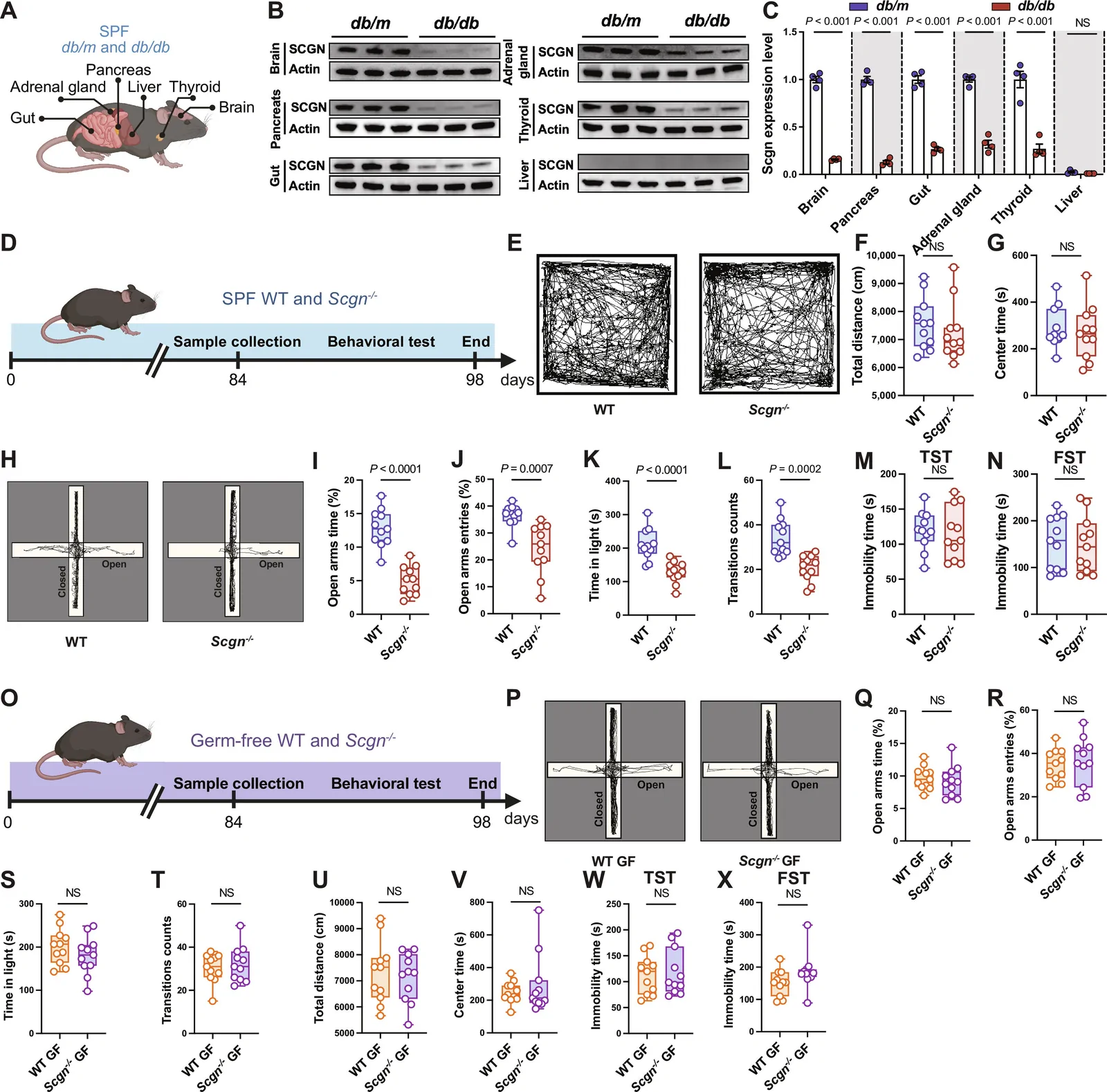
The diabetes-related gene SCGN might mediate anxiety-like behaviors via the microbiota. (A) The schematic diagram details the procedure for tissue sample collection from SPF *db/m* and *db/db* mice. The tissues collected include the gut, adrenal gland, pancreas, liver, thyroid, and brain. (B) Representative immunoblots illustrate the expression of SCGN in various tissues derived from *db/m* and *db/db* mice. (C) A quantitative analysis of SCGN expression levels across different tissues (*n* = 4 mice per group). (D) The experimental timeline for SPF WT and *Scgn*^−/−^ mice is detailed. Following weaning, the experimental mice were housed individually to eliminate social influences. Fecal, brain, and plasma samples were collected at 84 d of age to assess gut microbiota composition. During the subsequent 2 weeks (days 84 to 98), a comprehensive series of behavioral tests was performed in the following order: open-field test, elevated plus maze test, light–dark box test, tail suspension test, and forced swim test. Notably, a subset of mice was sacrificed immediately after the initial EPM test for c-Fos immunostaining to evaluate neuronal activation patterns. (E) Representative movement trajectories of SPF WT and *Scgn*^−/−^ mice during the open-field test are depicted. (F) The total distance traveled by SPF WT and *Scgn*^−/−^ mice in the open-field test is quantified (*n* = 11 mice per group). (G) The duration spent in the center area during the open-field test by SPF WT and *Scgn*^−/−^ mice is reported (*n* = 11 mice per group). (H) Representative movement trajectories for SPF WT and *Scgn*^−/−^ mice during the elevated plus maze test. (I) The duration spent in the open arms of the elevated plus maze test by SPF WT and *Scgn*^−/−^ mice is quantitatively assessed (*n* = 11 mice per group). (J) The number of entries into the elevated plus maze test by SPF WT and *Scgn*^−/−^ mice is documented (*n* = 11 mice per group). (K) The duration spent in the light compartment of the light–dark box test by SPF WT and *Scgn*^−/−^ mice is evaluated (*n* = 11 mice per group). (L) Counts of compartment transitions observed in SPF WT and *Scgn*^−/−^ mice during the light–dark box test (*n* = 11 mice per group). (M) The immobility time of SPF WT and *Scgn*^−/−^ mice during the tail suspension test is measured (*n* = 11 mice per group). (N) The immobility time of SPF WT and *Scgn*^−/−^ mice during the forced swim test is also documented (*n* = 11 mice per group). (O) This diagram presents the experimental timeline involving GF WT and *Scgn*^−/−^ mice. Following weaning, the mice were individually housed in separate cages. At 84 d of age, samples were obtained from the mPFC and plasma. Behavioral assessments were then conducted over the following 2 weeks in the subsequent order: open-field test, elevated plus maze test, light–dark box test, tail suspension test, and forced swim test. (P) Representative movement trajectories of GF WT and *Scgn*^−/−^ mice in the elevated plus maze test. (Q) The time spent in open arms by GF WT and *Scgn*^−/−^ mice in the elevated plus maze test is reported (*n* = 11 mice per group). (R) The number of entries into open arms by GF WT and *Scgn*^−/−^ mice in the elevated plus maze test is presented (*n* = 11 mice per group). (S) The duration spent in the light compartment by GF WT and *Scgn*^−/−^ mice in the light–dark box test is documented (*n* = 11 mice per group). (T) The counts of compartment transitions for GF WT and *Scgn*^−/−^ mice in the light–dark box test are specified (*n* = 11 mice per group). (U) The total distance traveled by GF WT and *Scgn*^−/−^ mice in the open-field test is noted (*n* = 11 mice per group). (V) The duration spent in the center area for GF WT and *Scgn*^−/−^ mice in the open-field test is indicated (*n* = 11 mice per group). (W) The immobility time of GF WT and *Scgn*^−/−^ mice during the tail suspension test is recorded (*n* = 11 mice per group). (X) The immobility time of GF WT and *Scgn*^−/−^ mice during the forced swim test is documented (*n* = 11 mice per group). Data are presented in box plots, showing the median and interquartile range, with individual data points representing biological replicates. For statistical analysis, an unpaired 2-tailed Student’s *t* test was applied to the data points labeled in (C), (F), (G), (I) to (N), and (Q) to (X). NS, not significant.

To examine diabetes-associated anxiety-like behaviors, we first conducted comprehensive behavioral assessments of 12-week-old *Scgn*^−/−^ mice and wild-type (WT) C57BL/6 controls (Fig. 1D). Open-field tests revealed comparable levels of locomotor activity and center zone occupancy duration between the 2 groups (Fig. 1E to G). However, *Scgn*^−/−^ mice exhibited pronounced anxiety-like behavioral phenotypes in both the elevated plus maze and light–dark box tests, manifested by significantly reduced time spent exploring open arms and fewer entries (Fig. 1H to J), a shorter duration in the light compartment (Fig. 1K), and a decreased number of intercompartment transitions (Fig. 1L). Conversely, the tail suspension and forced swim tests showed no significant differences in immobility time between the groups (Fig. 1M and N), indicating an absence of depression-like behavior. To validate the diabetes-associated anxiety phenotype, we conducted the same behavioral experiments in 2 additional diabetic models, high-fat-diet (HFD)-fed mice and db/db mice. Consistent with previous findings [28,29], both HFD-fed mice and db/db mice exhibited typical diabetes-related metabolic abnormalities, including hyperglycemia (Fig. S1E and F). Behavioral analyses using the open-field test, elevated plus maze, and light–dark box test showed that, similar to *Scgn*^−/−^ mice, both HFD-fed and db/db mice displayed anxiety-like phenotypes. Compared with WT mice, they made fewer entries into the open arms, showed fewer transitions into the light compartment, and spent less time in the open arms and the light compartment while displaying similar total locomotor distance in the open-field test (Fig. S1G to N). These findings indicate that the anxiety-like behaviors represent a consistent behavioral phenotype across distinct experimental models of diabetes.

On the other hand, cognitive function tests revealed no detectable deficits in *Scgn*^−/−^ mice (Fig. S2A to G). These findings suggest that SCGN plays a critical role in modulating anxiety-related behaviors but is not essential for maintaining cognitive performance.

Importantly, bulk RNA-sequencing analysis of colon tissue from specific pathogen-free (SPF) WT and *Scgn*^−/−^ mice revealed that the differentially expressed genes were significantly enriched in Gene Ontology terms related to intestinal antimicrobial defense, including antibacterial humoral response, antimicrobial humoral response mediated by antimicrobial peptides, and defense responses to both Gram-positive and Gram-negative bacteria (Fig. S2H to J). To elucidate the gut microbiota’s potential contribution to the anxiety-like phenotype, we generated germ-free (GF) *Scgn*^−/−^ mice and systematically evaluated their neurobehavioral profiles (Fig. 1O). Notably, GF *Scgn*^−/−^ mice exhibited no significant behavioral differences from GF WT controls across multiple anxiety-related parameters, including open-arms exploration time and entries (Fig. 1P to R), light compartment duration (Fig. 1S), and intercompartment transition frequency (Fig. 1T). Similarly, we detected no abnormalities in motor function (Fig. 1U and V) or depression-related behaviors (Fig. 1W and X). In contrast, *Scgn*^−/−^ mice maintained their autism-like phenotypes under GF conditions (Fig. S3A to H), indicating that the pathogenesis of autism-like behaviors in this model is independent of gut microbiota and involves mechanisms distinct from those underlying anxiety-related behaviors.

SCGN is a calcium sensor highly expressed in enteroendocrine cells and pancreatic β cells; its deficiency results in secretory defects rather than loss of hormone-producing cells. Consequently, in *Scgn*-deficient models, nutrient-stimulated glucagon-like peptide-1 (GLP-1) release and glucose-stimulated insulin secretion are blunted [21,30,31]. Enteroendocrine hormones, including GLP-1, peptide YY (PYY), cholecystokinin (CCK), and 5-hydroxytryptamine (5-HT), are key regulators of intestinal transit, bile-acid flow and signaling, and mucus secretion, collectively shaping the luminal niche and thus microbiota composition [32–34]. To test whether restoring gut microbiota could ameliorate the behavioral phenotypes of *Scgn*^−/−^ mice, we co-housed *Scgn*^−/−^ mice with WT mice (Fig. S4A). Subsequent behavioral analyses revealed a marked attenuation of anxiety-like behaviors in *Scgn*^−/−^ mice following microbiota restoration (Fig. S4B to G). Collectively, these findings indicate that the anxiety-like phenotypes observed in *Scgn*^−/−^ mice are primarily associated with alterations in the gut microbiota rather than arising solely from direct effects of SCGN deficiency.

In parallel with our observations in *Scgn*^−/−^ mice, we investigated the contribution of the gut microbiota to anxiety-like behaviors in *db/db* mice through a series of comprehensive behavioral tests (Fig. S5A). The open-field test revealed no significant differences in locomotor activity and center zone occupancy between *db/db* mice and *db/m* control mice (Fig. S5B to D). However, *db/db* mice exhibited pronounced anxiety-like behaviors in both the elevated plus maze (Fig. S5E to G) and light–dark box tests (Fig. S5H and I). Furthermore, the forced swim and tail suspension tests revealed no evidence of depression-like behaviors in this model (Fig. S5J and K). Notably, antibiotic intervention markedly ameliorated anxiety-like behaviors in *db/db* mice without affecting locomotor activity or depression-related behaviors (Fig. S5B to K). Because GF *db/db* mice were not examined, these findings demonstrated an association with microbiota depletion rather than with the complete absence of gut microbiota. Nevertheless, these results support a potential regulatory role for SCGN in the pathogenesis of diabetes and demonstrate that alterations in the gut microbiota contribute substantially to diabetes-associated anxiety-like behaviors.

### SCGN deficiency is associated with anxiety-like behavior and a reduction in butyrate levels due to gut microbiota dysbiosis

To further elucidate the gut microbiota alterations associated with the anxiety-like phenotype of *Scgn*^−/−^ mice, we performed shotgun metagenomic sequencing of fecal samples from *Scgn*^−/−^, WT, and co-housed mice. The results revealed genotype-associated shifts in microbial community composition. However, α-diversity analysis revealed no significant differences in microbial diversity between WT and *Scgn*^−/−^ mice, as assessed by the Shannon index (Fig. 2A). In contrast, principal components analysis (PCA) and taxonomic relative abundance analysis revealed a distinct separation in microbial community structures among the groups (Fig. 2B and C). Kyoto Encyclopedia of Genes and Genomes (KEGG)-based linear discriminant analysis effect size (LefSe) analysis of species-level relative abundance profiles identified butanoate (butyrate) metabolism as a key discriminatory pathway between the WT and *Scgn*^−/−^ mice, indicating significant differences in the predicted functional composition (Fig. 2D). Notably, microbiome analysis revealed no significant differences in microbial community composition between co-housed *Scgn*^−/−^ and WT mice, supporting effective normalization of the microbial community following co-housing (Fig. 2A to C). Consistently, a comparison of taxa associated with butyrate metabolism showed that *Odoribacter splanchnicus* (OD) displayed a significant difference between the groups, suggesting its potential contribution to the altered butyrate-related functional profile (Fig. 2E). Moreover, Spearman correlation heatmap associated with behavior revealed a significant negative correlation between OD and anxiety-related phenotypes (Fig. S5L), suggesting that greater abundance of this taxon is associated with attenuated anxiety-like behavior and highlighting its potential functional relevance.

**Fig. 2.**
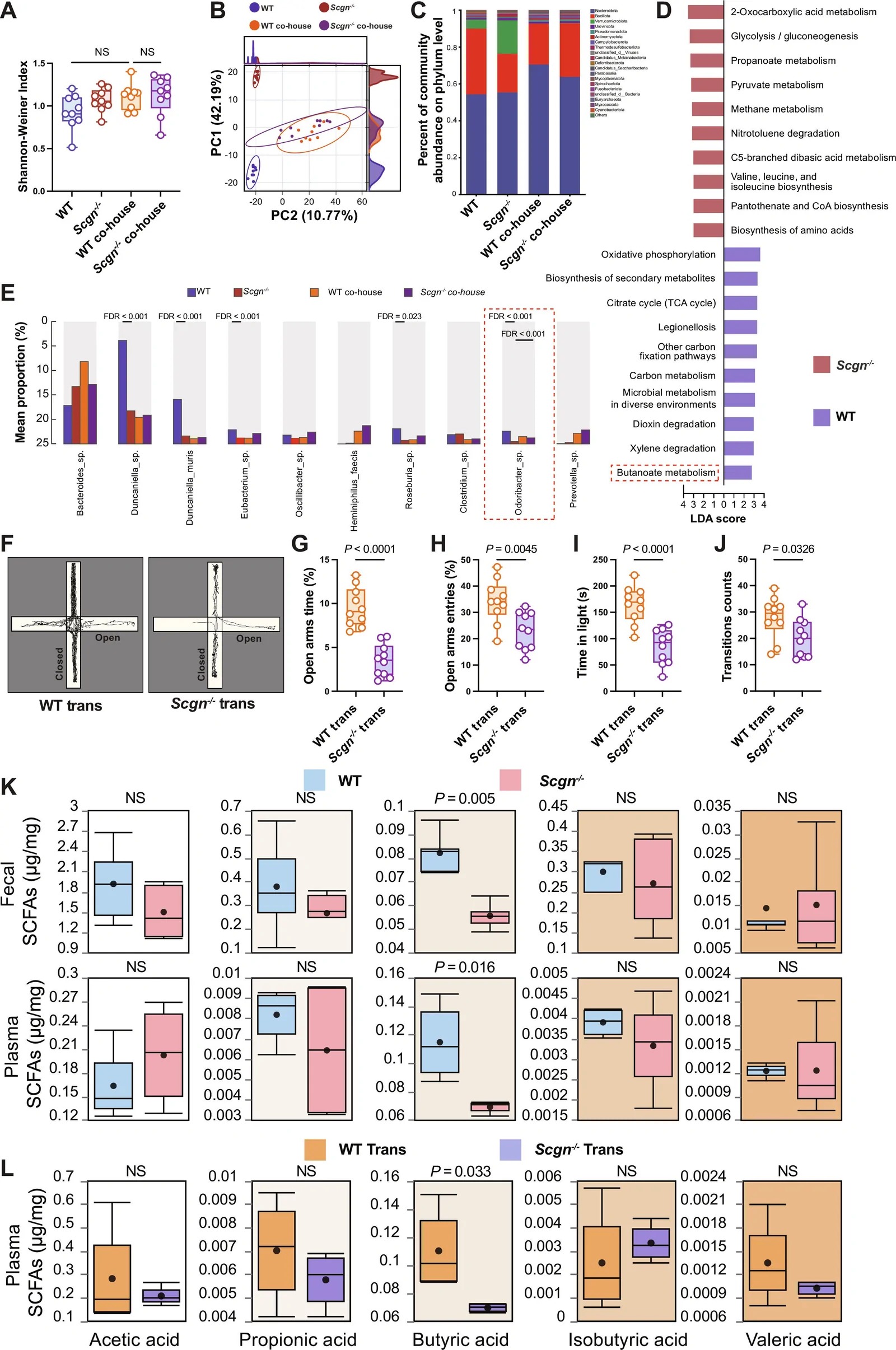
SCGN knockout alters gut microbiota composition and SCFA metabolism, while also induces anxiety-like behavior. (A) The α diversity of fecal microbiota in SPF WT, *Scgn*^−/−^, and co-housed mice was measured using the Shannon–Wiener index (*n* = 9 mice per group). (B) A PCA plot illustrating β diversity in gut microbiota communities among SPF WT, *Scgn*^−/−^, and co-housed mice (*n* = 9 mice per group). (C) Relative abundance profiles at the phylum level for SPF WT, *Scgn*^−/−^, and co-housed mice are shown (*n* = 9 mice per group). (D) Differentially enriched KEGG pathways inferred from the microbiome are depicted, with pathways enriched in *Scgn*^−/−^ mice indicated in red, and those enriched in WT mice represented in blue. (E) The mean relative abundance (%, mean proportion) of representative taxa that are differentially abundant across the 4 groups is displayed. The dashed box highlights *Odoribacter splanchnicus* (*n* = 9 mice per group). (F) Representative movement trajectories of GF mice following FMT from SPF WT and *Scgn*^−/−^ donors in the elevated plus maze test are provided. (G) The time spent in the open arms of GF mice following FMT from SPF WT and *Scgn*^−/−^ donors in the elevated plus maze test is reported (*n* = 10 mice per group). (H) Open arm entries of GF mice following FMT from SPF WT and *Scgn*^−/−^ donors in the elevated plus maze test (*n* = 10 mice per group). (I) The time spent in the light compartment of GF mice following FMT from SPF WT and *Scgn*^−/−^ donors in the light–dark box test is detailed (*n* = 10 mice per group). (J) Compartment transition counts of GF mice following FMT from SPF WT and *Scgn*^−/−^ donors in the light–dark box test are illustrated (*n* = 10 mice per group). (K) Levels of SCFAs measured in feces and plasma samples from SPF WT and SPF *Scgn*^−/−^ mice are provided (*n* = 4 mice per group). (L) Levels of SCFAs measured in plasma samples from mice following FMT from SPF WT and *Scgn*^−/−^ donors (*n* = 4 mice per group). Data are presented as box plots denoting the median and interquartile range, with individual data points representing biological replicates. NS, not significant. Statistical analysis: A one-way ANOVA was conducted, followed by Tukey’s post hoc test for comparisons in (A) and (E). An unpaired 2-tailed Student’s *t* test was employed for the analysis of (G) to (L).

To directly evaluate the behavioral consequences of these microbial alterations, we performed FMT into GF WT mice. Recipients of the microbiota from *Scgn*^−/−^ donors exhibited more pronounced anxiety-like behaviors than recipients of the WT microbiota (Fig. 2F), as evidenced by reduced open-arm exploration time (Fig. 2G), a lower open-arm entries in the elevated plus maze (Fig. 2H), decreased time spent in the light compartment duration (Fig. 2I), and a lower frequency of transitions in the light–dark box tests (Fig. 2J). These findings indicate that SCGN deficiency-associated microbiota dysbiosis substantially contributes to anxiety-like phenotypes.

We next quantified short-chain fatty acids (SCFAs) in fecal and plasma samples from *Scgn*^−/−^ and WT mice (Fig. 2K). Among the 5 detectable SCFAs, butyrate levels were significantly lower in *Scgn*^−/−^ mice than in WT controls. Conversely, the concentrations of acetic acid, propionic acid, isobutyric acid, and valeric acid were comparable between the 2 groups. Consistently, GF recipients of the *Scgn*^−/−^ donor microbiota also exhibited significantly reduced plasma butyrate levels compared with recipients of WT microbiota (Fig. 2L). Collectively, these results suggest that SCGN deficiency may influence anxiety-like behaviors through alterations in gut microbial composition and SCFA metabolism, indicating a potential gut–brain axis mechanism underlying the behavioral phenotype.

### SCGN-deficient diabetic mice exhibit abnormal tryptophan metabolism in the mPFC

To investigate the mechanistic basis of the anxiety-like phenotypes associated with SCGN deficiency, we performed c-Fos immunostaining across multiple anxiety-related brain regions (Fig. 3A). c-Fos is an immediate early gene product and a widely used marker of neuronal activation that is rapidly induced following behavioral stimulation, allowing region-specific neuronal activity to be visualized [35]. Under resting conditions, c-Fos expression was low and comparable between the 2 groups across all examined regions (Fig. S6A and B). However, following exposure to the elevated plus maze (EPM) test, *Scgn*^−/−^ mice exhibited a significant increase in c-Fos expression specifically in the mPFC compared with WT controls (Fig. 3A and B). In contrast, no apparent differences were observed in other established anxiety-related brain regions, including the paraventricular nucleus (PVN), hippocampus, and basolateral amygdala (BLA) (Fig. 3A and B). These findings indicate that the mPFC may act as a prominent site of neural dysregulation associated with anxiety-like phenotypes observed in these animals.

**Fig. 3.**
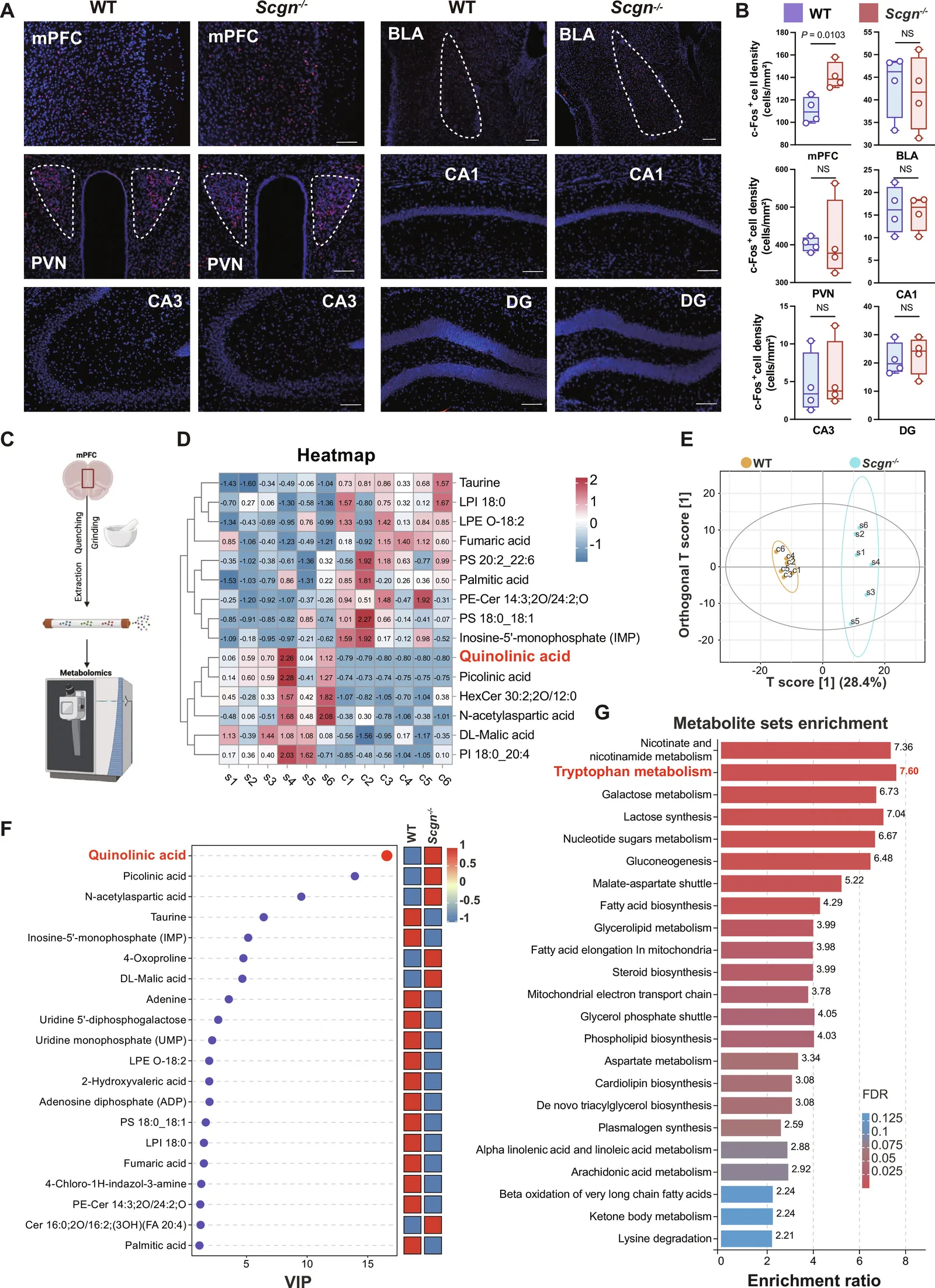
Molecular expression profiles and metabolomic analysis in *Scgn*^−/−^ mouse brain. (A) Representative images of c-Fos immunofluorescence in the mPFC, BLA, PVN, and the hippocampal CA1, CA3, and dentate gyrus (DG) regions of SPF WT and *Scgn*^−/−^ mice are shown at 90 min following the EPM test. Red: c-Fos; blue: 4′,6-diamidino-2-phenylindole (DAPI). Scale bars are 200 μm for the BLA and 100 μm for the remaining regions. White dashed lines delineate the boundaries of the analyzed brain regions (mPFC, BLA, PVN, CA1, CA3, and DG). (B) A comparison of the number of c-Fos-positive cells across various brain regions in SPF WT and *Scgn*^−/−^ mice is provided (*n* = 4 mice per group). (C) An untargeted metabolomic analysis of the mPFC was conducted in SPF WT and *Scgn*^−/−^ mice. (D) The heatmap illustrates the differential metabolites identified in the mPFC of SPF WT and *Scgn*^−/−^ mice (*n* = 6 mice per group) (C, SPF WT; S, SPF *Scgn*^−/−^). (E) A PCA plot depicts the metabolic profiles in the mPFC of SPF WT and *Scgn*^−/−^ mice (*n* = 6 mice per group) (C, SPF WT; S, SPF *Scgn*^−/−^). (F) The top 15 differential metabolites are ranked based on their VIP values when comparing SPF WT and *Scgn*^−/−^ mice. (G) An MSEA was performed on all differential expression metabolites between SPF WT and *Scgn*^−/−^ mice. Data are presented as box plots showing the median and interquartile range, with individual data points representing biological replicates. NS, not significant. Statistical analysis: An unpaired 2-tailed Student’s *t* test was employed for the analysis in (B).

We next performed metabolomics profiling of the mPFC (Fig. 3C and E), which showed a significant elevation of QA in *Scgn*^−/−^ mice (Fig. 3D). Among the metabolites analyzed, QA exhibited the highest variable importance in projection (VIP) score (Fig. 3F). Metabolite set enrichment analysis (MSEA) consistently identified tryptophan metabolism, a pathway directly linked to QA biosynthesis, as the most significantly enriched pathway (Fig. 3G). Butyrate has been reported to inhibit indoleamine 2,3-dioxygenase 1 (IDO1), a critical enzyme in tryptophan metabolism and QA production [36,37]. QA functions as an *N*-methyl-d-aspartic acid (NMDA) receptor agonist and can enhance neuronal excitability, which may contribute to anxiety-like behaviors (Fig. 4A). Therefore, we examined the expressions of correlated key enzymes in the mPFC. Protein analyses revealed a significant up-regulation of IDO1 and kynureninase (KYNU) expression in the mPFC of *Scgn*^−/−^ mice compared with WT controls (Fig. 4B to G). Consequently, the concentrations of l-kynurenine (Fig. 4H) and QA (Fig. 4I) were markedly elevated in the mPFC of *Scgn*^−/−^ mice.

**Fig. 4.**
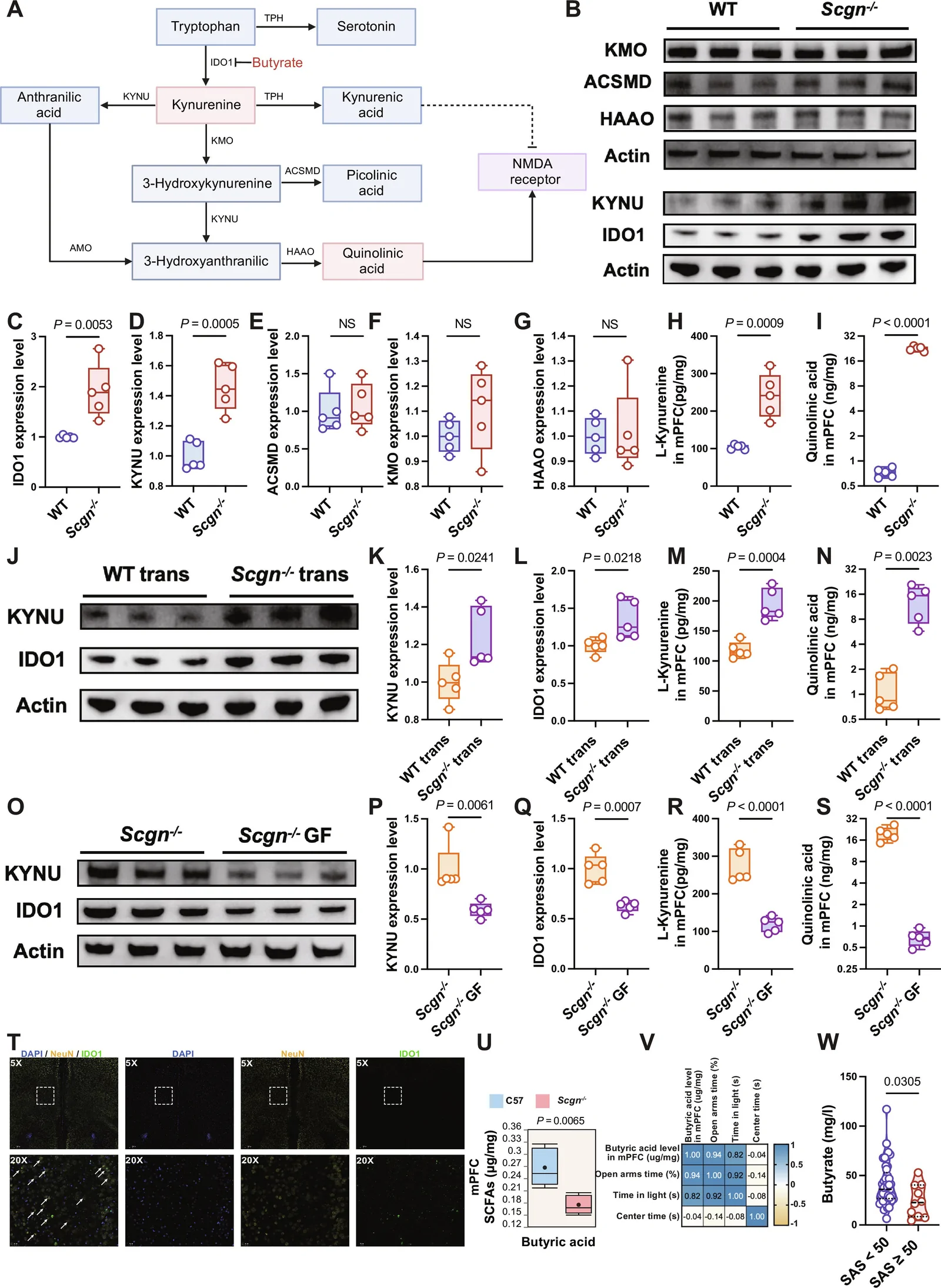
Altered expression of tryptophan metabolism enzymes in the mPFC and associated metabolite profiles. (A) Schematic illustration depicting the modulation of tryptophan metabolism in the brain by butyrate. (B) Representative Western blots showing the expression levels of tryptophan metabolism-related enzymes in the mPFC of SPF WT and *Scgn*^−/−^ mice. (C to G) Protein expression levels of enzymes related to tryptophan metabolism in SPF WT and *Scgn*^−/−^ mice are displayed, specifically (C) IDO1, (D) KYNU, (E) ACMSD, (F) KMO, and (G) HAAO (*n* = 5 mice per group). (H and I) Concentrations of l-kynurenine and QA in the mPFC of SPF WT and *Scgn*^−/−^ mice (*n* = 5 mice per group). (J) Representative Western blots of KYNU and IDO1 in the mPFC of GF mice following FMT from SPF WT and *Scgn*^−/−^ donors. (K and L) Protein expression levels of KYNU and IDO1 in GF mice post-FMT from SPF WT and *Scgn*^−/−^ donors (*n* = 5 mice per group). (M and N) Concentrations of l-kynurenine and QA in GF mice following FMT from SPF WT and *Scgn*^−/−^ donors in the mPFC (*n* = 5 mice per group). (O) Representative Western blots of KYNU and IDO1 in the mPFC of both SPF and GF *Scgn*^−/−^ mice. (P and Q) Protein expression levels of KYNU and IDO1 in SPF and GF *Scgn*^−/−^ mice (*n* = 5 mice per group). (R and S) Concentrations of l-kynurenine and QA in the mPFC of SPF and GF *Scgn*^−/−^ mice (*n* = 5 mice per group). (T) Immunofluorescent staining in the mPFC illustrates the presence of IDO1 (green), NeuN (yellow), and DAPI (blue). The left panels display images at 5× magnification (scale bar, 100 μm), while the right panels display images at 20× magnification (scale bar, 25 μm) (*n* = 3 mice per group). (U) Butyric acid levels in the mPFC of SPF WT and *Scgn*^−/−^ mice are noted, with a sample size of *n* = 4 mice per group. (V) A correlation heatmap illustrates the relationship between butyric acid levels in the mPFC and various behavioral indices. Notably, butyric acid content is positively correlated with open arm time (%) in the elevated plus maze and time spent in light (seconds) in the light/dark box test. However, it does not show a significant correlation with center time (seconds). The color scale indicates Pearson correlation coefficients (*r*), with blue indicating positive and yellow indicating negative correlations. (W) Plasma butyrate levels of diabetic patients with SAS < 50 (*n* = 41) and SAS ≥ 50 (*n* = 10). Data are presented as box plots, showing the median and interquartile range; individual data points represent biological replicates. NS, not significant. Statistical analysis: An unpaired 2-tailed Student’s *t* test was applied to comparisons: (C) to (I), (K) to (N), (P) to (S), (U), and (W). Spearman correlation with linear regression fitting was used for (V).

To determine whether SCGN deficiency-associated gut microbiota dysbiosis affected mPFC tryptophan metabolism, we transplanted fecal microbiota from *Scgn*^−/−^ donors into GF WT recipients. The recipients exhibited significantly increased IDO1 and KYNU protein levels (Fig. 4J to L), accompanied by elevated concentrations of l-kynurenine and QA in the mPFC relative to controls (Fig. 4M and N). Conversely, the GF derivation of *Scgn*^−/−^ mice led to a significant reduction in both IDO1 and KYNU protein levels in the mPFC (Fig. 4O to Q) and normalized l-kynurenine and QA levels to those observed in GF WT controls (Fig. 4R and S), whereas no significant differences were detected between the 2 GF groups for these measures (Fig. S7A to E). Importantly, similar dysregulation of the IDO1–KYNU–QA pathway was observed in additional diabetic models exhibiting anxiety-like phenotypes. Compared with WT controls, both HFD-fed and db/db mice showed significantly increased levels of IDO1 and KYNU, elevated levels of l-kynurenine and QA, and reduced butyrate levels in mPFC (Fig. S7F to K). Therefore, these findings indicate that the gut microbiota–butyrate–tryptophan metabolism–mPFC axis is involved in diabetes-associated neurobehavioral alterations.

To determine the cellular source of IDO1 in the mPFC, we conducted in situ hybridization for IDO1 and immunofluorescence (IF) staining for neuronal nuclei antigen (neurons, NeuN), glial fibrillary acidic protein (astrocytes, GFAP), and ionized calcium binding adapter molecule 1 (microglia, IBA1). IDO1 was predominantly localized to NeuN^+^ neurons (79.1%), with a smaller fraction detected in GFAP^+^ astrocytes (14%), and virtually no detectable expression in IBA1^+^ microglia (0%) (Fig. 4T and Fig. S7L to N). Collectively, these data indicate that neurons represent the predominant cellular source of IDO1 in the mPFC under the conditions examined.

Consistent with our analyses of ‌feces and plasma samples, gas chromatography–mass spectrometry (GC/MS) revealed significantly reduced butyrate levels in the mPFC of *Scgn*^−/−^ mice compared with WT controls (Fig. 4U). In contrast, acetic acid and 4-methylvaleric acid levels remained unchanged (Fig. S7O). Moreover, mPFC butyrate levels showed a strong correlation with anxiety-related behavioral measures, displaying robust positive associations with open-arms exploration in the elevated plus maze and time spent in the light compartment (Fig. 4V). To validate these findings, we examined circulating butyrate levels in 51 patients with diabetes stratified by anxiety status. Self-rating anxiety scale (SAS) was used to assess the anxiety status of patients. The anxiety (SAS ≥ 50, *n* = 10) and non-anxiety (SAS < 50, *n* = 41) groups were comparable in age (61.70 ± 9.01 years versus 57.05 ± 10.09 years) and diabetes duration (7.26 ± 5.32 years versus 10.03 ± 8.05 years). We found that plasma butyrate levels were significantly higher in patients with SAS scores <50 than those with SAS scores ≥50 (Fig. 4W), indicating that reduced systemic butyrate levels are associated with anxiety comorbidity in diabetes. These findings associate central butyrate levels with anxiety-like behaviors through restoration of mPFC tryptophan metabolism.

To directly test whether elevated mPFC QA is sufficient to induce anxiety-like behaviors, we performed stereotaxic injection of QA into the mPFC of WT mice. QA treatment mice exhibited marked anxiety-like behavior, including reduced open arm and light compartment exploration (Fig. S8F to J), without affecting glucose tolerance or general locomotion (Fig. S8A to E), indicating that mPFC QA accumulation (Fig. S8K) is sufficient to induce anxiety-like phenotypes. Crucially, correlation analysis across WT and *Scgn*^−/−^ mice, housed either separately or in co-housing, revealed a significant positive association between mPFC QA levels and anxiety severity (Fig. S8L).

Collectively, these findings demonstrate that in diabetic models, SCGN deficiency-associated gut microbiota dysbiosis reduces butyrate availability, which is associated with increased IDO1 and KYNU levels in mPFC neurons and subsequent pathological accumulation of QA.

### Butyrate alleviates anxiety-like behaviors and aberrant tryptophan metabolism in the mPFC of SCGN-deficient mice

To directly evaluate the therapeutic potential of butyrate, we administered sodium butyrate to both *Scgn*^−/−^ and WT mice (Fig. 5A) at 5 g/l, based on an initial dose-selection experiment (Fig. S9A to D). Butyrate treatment significantly ameliorated anxiety-like behaviors in *Scgn*^−/−^ mice, as evidenced by increased open arm exploration time (Fig. 5B and C) and the percentage of open arm entries in the elevated plus maze (Fig. S9E), increased time spent in the light compartment (Fig. 5D), and more intercompartment transitions in the light–dark tests (Fig. S9F). Mechanistically, butyrate treatment effectively alleviated abnormal tryptophan metabolism in the mPFC of *Scgn*^−/−^ mice by reducing IDO1 and KYNU protein levels (Fig. 5E to G) and decreasing l-kynurenine and QA (Fig. 5H and I). Metabolomic profiling (Fig. 5J and K) confirmed a significant reduction of QA levels in the mPFC of butyrate-treated *Scgn*^−/−^ mice (Fig. 5L), with QA exhibiting the highest VIP score among the metabolites analyzed (Fig. 5M). MSEA identified tryptophan metabolism as the most significantly enriched pathway (Fig. 5N).

**Fig. 5.**
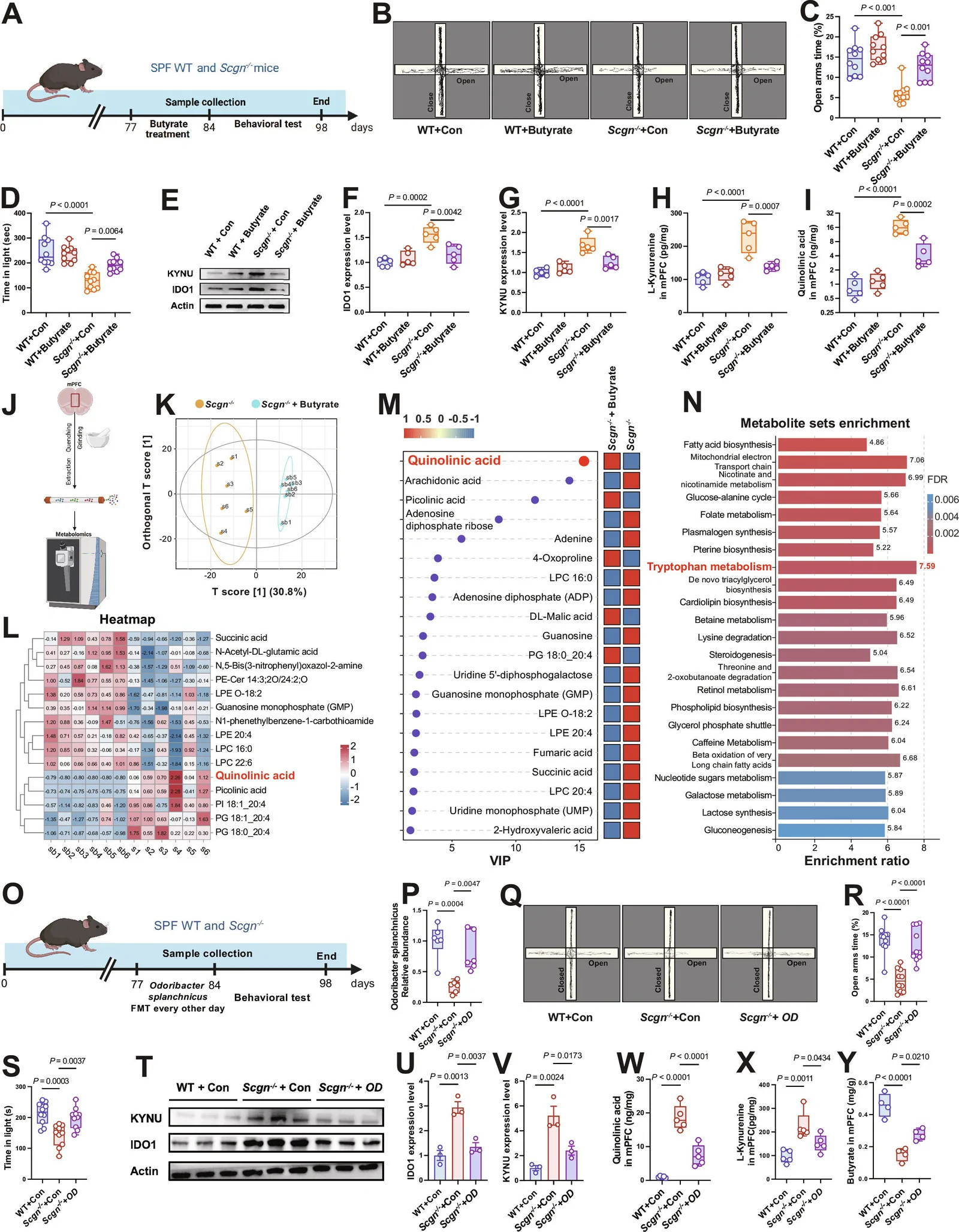
Butyrate supplement and *Odoribacter splanchnicus* treatment influence behavioral phenotypes and metabolomic changes in the mPFC of *Scgn*^−/−^ mice. (A) The experimental timeline illustrates the administration of butyrate to SPF WT and *Scgn*^−/−^ mice. Following weaning, the mice were individually housed in separate cages. At 77 d of age, the experimental mice underwent 1 week of butyrate treatment, during which their drinking water was substituted with a solution containing 5 g/l butyrate. Subsequent to treatment, brain samples from the mPFC were collected at 84 d of age, and behavioral tests were conducted over the ensuing 2 weeks in the following sequence: elevated plus maze test and light–dark box test. (B) Representative movement trajectories illustrate the behavior of both butyrate-treated and untreated SPF WT and *Scgn*^−/−^ mice during the elevated plus maze test. (C) The duration spent in the open arms of the elevated plus maze test is presented, comparing butyrate-treated and untreated SPF WT and *Scgn*^−/−^ mice (*n* = 10 mice per group). (D) The duration spent in the light compartment during the light–dark box test is reported for both butyrate-treated and untreated SPF WT and *Scgn*^−/−^ mice (*n* = 10 mice per group). (E) Representative Western blots depict the expression of enzymes associated with tryptophan metabolism in the mPFC of both butyrate-treated and untreated SPF WT and *Scgn*^−/−^ mice. (F and G) The protein expression levels of KYNU (F) and IDO1 (G) were evaluated in both butyrate-treated and untreated SPF WT and *Scgn*^−/−^ mice (*n* = 5 mice per group). (H and I) The concentrations of QA (H) and l-kynurenine (I) in the mPFC of butyrate-treated and untreated SPF WT and *Scgn*^−/−^ mice were also evaluated (*n* = 5 mice per group). (J) An untargeted metabolomics analysis of the mPFC from butyrate-treated and untreated SPF *Scgn*^−/−^ mice was conducted (*n* = 6 mice per group). (K) A PCA plot illustrates the metabolic profiles in the mPFC of butyrate-treated and untreated SPF *Scgn*^−/−^ mice (*n* = 6 mice per group) (S, SPF *Scgn*^−/−^; SB, SPF *Scgn*^−/−^ butyrate-treated). (L) A representative heatmap shows the differential metabolites in the mPFC of butyrate-treated and untreated SPF *Scgn*^−/−^ mice (*n* = 6 mice per group) (S, SPF *Scgn*^−/−^; SB, SPF *Scgn*^−/−^ butyrate-treated). (M) The top 15 differential metabolites, ranked VIP values, are compared between butyrate-treated and untreated SPF *Scgn*^−/−^ mice. (N) An MSEA of all differential metabolites between butyrate-treated and untreated SPF *Scgn*^−/−^ mice was performed. (O) The experimental schedule for SPF WT and *Scgn*^−/−^ mice is detailed below. After weaning, laboratory mice were housed separately to eliminate social influence. At 77 d of age, FMT followed by monobacterial treatment was administered, and at 84 d, fecal, brain, and plasma samples were stopped to assess gut microbiome composition. Over the following 2 weeks (days 84 to 98), a series of comprehensive behavioral tests were conducted in the following order: open-field test, elevated plus maze test, light and dark box test, tail dangling test, and forced swimming test. (P) Relative abundance of *Odoribacter splanchnicus* in the gut of mice with different genotypes. Compared with WT mice, *Scgn*^−/−^ mice showed significantly reduced relative abundance of *Odoribacter splanchnicus*. OD intervention in *Scgn*^−/−^ mice significantly restored the abundance of *Odoribacter splanchnicus* compared with the untreated *Scgn*^−/−^ group. Data are presented as box plots with individual data points overlaid (*n* = 6 mice per group). (Q) Representative movement trajectories illustrate the behavior of both SPF WT + con, *Scgn*^−/−^ + con, and *Scgn*^−/−^ + OD mice during the elevated plus maze test. (R) The duration spent in the open arms of the elevated plus maze test is presented, comparing SPF WT + con, *Scgn*^−/−^ + con, and *Scgn*^−/−^ + OD mice (*n* = 10 mice per group). (S) The duration spent in the light compartment during the light–dark box test is reported for SPF WT + con, *Scgn*^−/−^ + con, and *Scgn*^−/−^ + OD mice (*n* = 10 mice per group). (T) Representative Western blots of KYNU and IDO1 expression in the mPFC of SPF WT + con, *Scgn*^−/−^ + con, and *Scgn*^−/−^ + OD mice. (U and V) Protein expression levels of KYNU and IDO1 were evaluated in SPF WT + con, *Scgn*^−/−^ + con, and *Scgn*^−/−^ + OD mice (*n* = 3 mice per group). (W and X) The concentrations of QA (W), l-kynurenine (X), and butyrate (Y) in the mPFC of SPF WT + con, *Scgn*^−/−^ + con, and *Scgn*^−/−^ + OD mice (*n* = 5 mice per group). (Y) Concentrations of butyrate in the mPFC of SPF WT + con, *Scgn*^−/−^ + con, and *Scgn*^−/−^ + OD mice (*n* = 4 mice per group). Data are presented as box plots, showing the median and interquartile range, with individual data points representing biological replicates. NS, not significant. Statistical analysis: A one-way ANOVA was conducted, followed by Tukey’s post hoc test for comparisons in (C), (D), (F) to (I), (P), (R), (S), and (U) to (Y).

To verify whether the therapeutic effects of butyrate depended on the gut microbiota, we conducted FMT from *Scgn*^−/−^ or WT donors into antibiotic-pretreated WT recipients (Fig. S9G), followed by butyrate administration. Recipients of *Scgn*^−/−^ microbiota exhibited anxiety-like behaviors, accompanied by dysregulation of mPFC proteins and metabolites, all of which were significantly ameliorated by butyrate supplementation (Fig. S9H to Q). To further substantiate these findings and exclude potential confounding effects of antibiotics, we performed parallel experiments in GF WT recipients. FMT from *Scgn*^−/−^ donors similarly induced anxiety-like behaviors and activation of the mPFC kynurenine pathway (KP), characterized by increased IDO1/KYNU expression and elevated levels of l-kynurenine and QA. Butyrate supplementation appeared to partially ameliorate the behavioral and metabolic abnormalities in these recipients (Fig. S10A to K).

To identify a specific bacterial species that could account for the butyrate-mediated anxiolytic effects, we colonized *Scgn*^−/−^ mice with *Odoribacter splanchnicus* (OD) via oral gavage (Fig. 5O). We validated successful colonization by species-specific quantitative polymerase chain reaction (PCR) of fecal DNA (Fig. 5P). IPGTT showed that OD treatment did not improve glucose intolerance (Fig. S11A and B), and open-field testing revealed no changes in general locomotor activity (Fig. S11C to E). Notably, OD colonization significantly ameliorated anxiety-like behaviors, as demonstrated by increased open arm exploration time (Fig. 5Q and R), increased open arm entries in the elevated plus maze (Fig. S11F), increased time spent in the light compartment (Fig. 5S), and more intercompartment transitions in the light–dark tests (Fig. S11G). Mechanistically, OD administration normalized dysregulated tryptophan metabolism in the mPFC by suppressing aberrant IDO1 and KYNU protein expression (Fig. 5T to V) and decreasing the elevated QA and l-kynurenine levels (Fig. 5W and X). Meanwhile, OD colonization increased butyrate concentrations in the mPFC (Fig. 5Y) of *Scgn*^−/−^ mice, as well as in feces and plasma (Fig. S11H and I).

Collectively, these findings illustrate that butyrate effectively alleviates anxiety-like behaviors associated with gut microbiota dysbiosis in *Scgn*^−/−^ mice. This therapeutic effect is primarily mediated by normalization of tryptophan metabolism and subsequent QA production in the mPFC.

### Butyrate alleviates anxiety by suppressing the hyperexcitability of pyramidal neurons in the mPFC of SCGN-deficient mice

The precise mechanisms by which abnormally elevated QA alters mPFC neuronal activity and promotes anxiety-like phenotypes remain incompletely understood. We therefore sought to determine whether butyrate could improve QA-induced neuronal dysfunction. To address this question, we implanted electrodes into the mPFC and recorded neuronal activity during elevated plus maze testing (Fig. 6A). Resting-state power spectral density (PSD) analysis of local field potentials (LFPs) across multiple frequency bands showed no significant difference between *Scgn*^−/−^ and WT mice (Fig. 6B). However, *Scgn*^−/−^ mice exhibited a significant increase in θ-band PSD specifically upon entry into the open arms, which was markedly attenuated by butyrate treatment (Fig. 6B). Spike sorting classified the recorded units into putative interneurons and pyramidal neurons (Fig. 6C and D). Analysis of neuronal firing rates within 3 s during the approach to the open arms revealed significantly increased activity among the putative pyramidal neurons in *Scgn*^−/−^ mice (Fig. 6E and F), while no significant differences were observed in putative interneuron activity (Fig. S12A and B). Notably, butyrate treatment effectively suppressed the pyramidal neuron hyperexcitability, indicating that mPFC pyramidal neurons represent the predominant pathological target of QA.

**Fig. 6.**
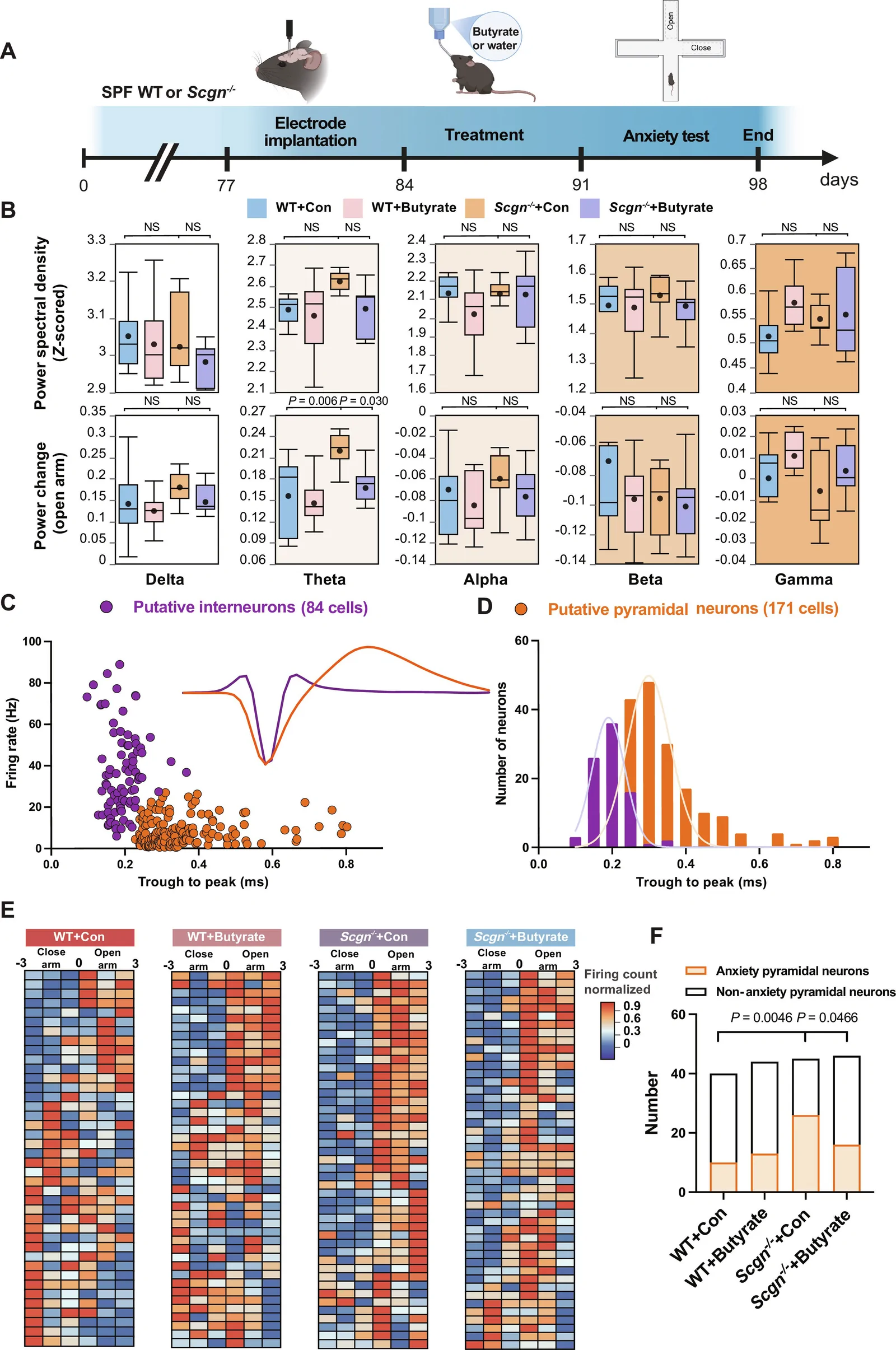
Effects of butyrate on neuronal activity in the mPFC of mice. (A) The experimental timeline diagram illustrates the in vivo *e*lectrophysiology. Following weaning, mice were individually housed. At 77 d of age, electrodes were surgically implanted in the mPFC. Beginning on day 84, the mice received a 1-week butyrate treatment at 5 g/l dissolved in their drinking water. Neuronal activity was subsequently recorded during the elevated plus maze test from days 91 to 98. Throughout this process, all mice were maintained in single-housing post-implantation to safeguard the headmounts; this housing condition was consistent across all experimental groups. (B) The PSD of LFPs in the mPFC was analyzed across various frequency bands, including delta, theta, alpha, beta, and gamma, for both butyrate-treated and untreated SPF WT and *Scgn*^−/−^ mice. The upper row presents basal levels, while the lower row depicts power fluctuations within 3 s before and after entry into the open arm during the elevated plus maze test (*n* = 9 to 10 mice per group). (C) Putative interneurons (84 cells, represented in purple) and putative pyramidal neurons (171 cells, represented in orange) were classified using *k*-means clustering based on metrics including spike trough-to-peak duration and firing rate. (D) A histogram illustrates the distribution of trough-to-peak durations for each neuronal subtype under investigation. (E) Heatmaps illustrate the normalized firing rates of pyramidal neurons in both butyrate-treated and untreated SPF WT and *Scgn*^−/−^ mice during the 3 s preceding and following transitions into closed and open arms across various experimental groups (*n* = 28 to 31 cells from 9 to 10 mice per group). (F) The quantification of anxiety-related pyramidal neurons in both butyrate-treated and untreated SPF WT and *Scgn*^−/−^ mice is also provided (*n* = 28 to 31 cells from 9 to 10 mice per group). The data are presented as box plots, showing the median and interquartile range, with individual data points corresponding to biological replicates. NS, not significant. Statistical analysis: A one-way ANOVA followed by Tukey’s post hoc test was employed for (B). Fisher’s exact test was utilized for (F).

To gain a deeper mechanistic insight, we performed whole-cell patch-clamp recordings from mPFC pyramidal neurons, measuring spontaneous excitatory postsynaptic currents (sEPSCs), inhibitory postsynaptic currents (sIPSCs), and action potential firing (Fig. 7A). *Scgn*^−/−^ mice demonstrated a significant increase in action potential firing, as evidenced by the higher number of APs evoked by current injection and the elevated spontaneous firing frequency (Fig. 7B and C), suggesting enhanced intrinsic excitability of the pyramidal neurons. Butyrate treatment significantly reduced the abnormally high action potential firing frequency in *Scgn*^−/−^ mice. Analysis of sEPSC (Fig. 7D) and sIPSC (Fig. S12C) revealed a notable increase in sEPSC frequency in *Scgn*^−/−^ mice (Fig. 7G and H). Conversely, sEPSC amplitude (Fig. 7E and F) or sIPSC amplitude (Fig. S12D and E) and frequency (Fig. S12F and G) were unaltered. To investigate the functional consequences of altered butyrate–IDO1–tryptophan pathway signaling, pharmacological modulators were applied during acute slice incubation to examine their effects on sEPSCs (Fig. 7I). Both sodium butyrate and the selective IDO1 inhibitor epacadostat reduced the elevated sEPSC frequency in *Scgn*^−/−^ mice toward WT levels. Conversely, direct QA infusion in WT slices recapitulated the increased sEPSC frequency observed in *Scgn*^−/−^ mice, without affecting sEPSC amplitude (Fig. 7J to M). These findings provide functional evidence linking the butyrate–IDO1–QA axis to enhanced excitatory synaptic transmission in mPFC pyramidal neurons.

**Fig. 7.**
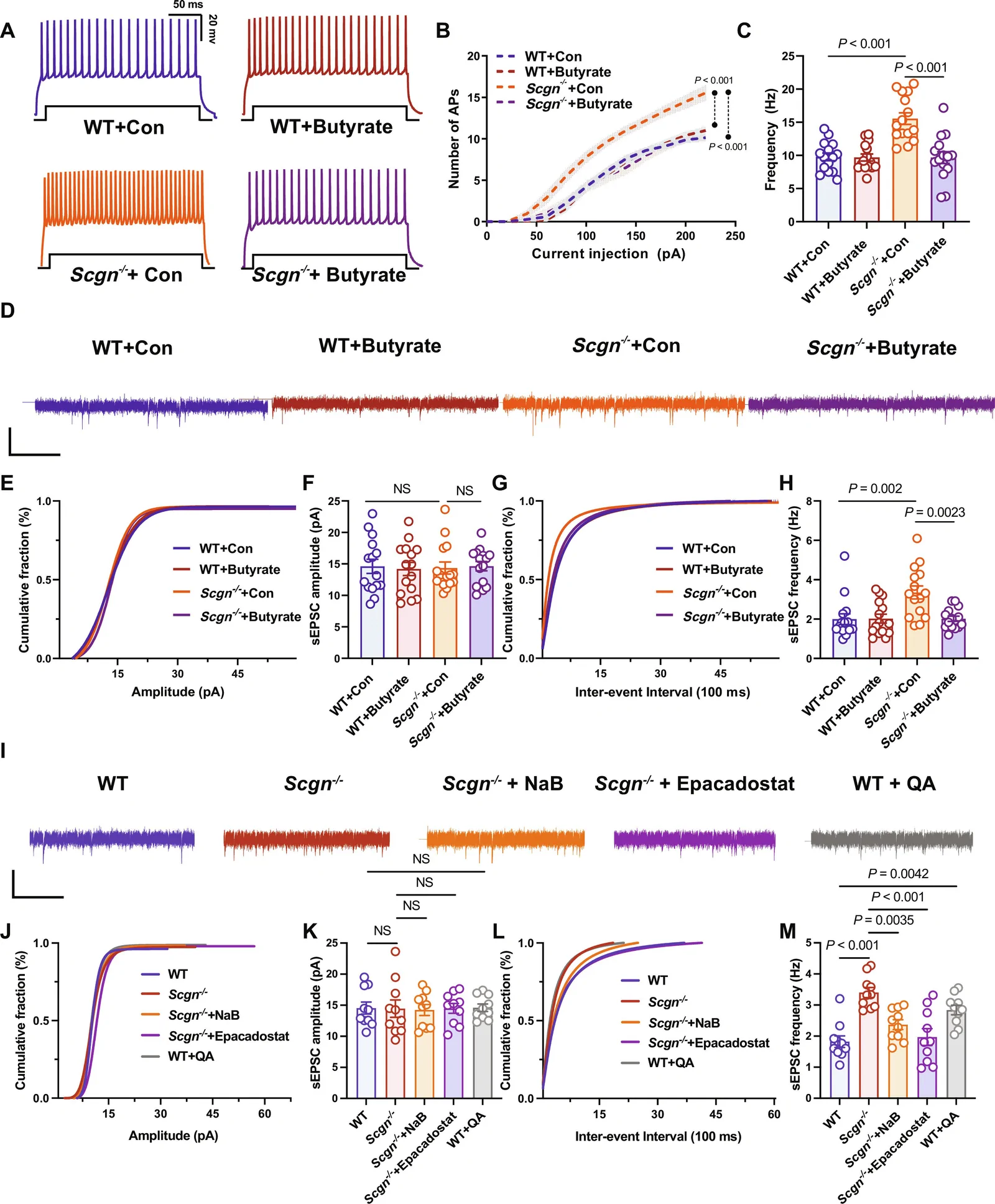
Effects of butyrate on pyramidal neuron excitability in vitro. (A) Representative action potential traces illustrating neuronal firing in butyrate-treated and untreated SPF WT and *Scgn*^−/−^ mice. (B) Quantification of the number of action potentials evoked by increasing current injections in butyrate-treated and untreated SPF WT and *Scgn*^−/−^ mice (*n* = 15 cells from 5 mice per group). (C) Neuronal firing frequency in response to a 200-pA current injection in butyrate-treated and untreated SPF WT and *Scgn*^−/−^ mice (*n* = 15 cells from 5 mice per group). (D) Representative traces of sEPSCs recorded from butyrate-treated and untreated SPF WT and *Scgn*^−/−^ mice. Scale bars: 500 ms (horizontal), 20 pA (vertical). (E) Cumulative distribution of sEPSC amplitudes recorded in pyramidal neurons from butyrate-treated and untreated SPF WT and *Scgn*^−/−^ mice (*n* = 15 cells from 5 mice per group). (F) Mean sEPSC amplitudes in pyramidal neurons from butyrate-treated and untreated SPF WT and *Scgn*^−/−^ mice (*n* = 15 cells from 5 mice per group). (G) Cumulative distribution of inter-event intervals of sEPSCs in pyramidal neurons from both butyrate-treated and untreated SPF WT and *Scgn*^−/−^ mice (*n* = 15 cells from 5 mice per group). (H) Mean sEPSC frequency in pyramidal neurons from butyrate-treated and untreated SPF WT and *Scgn*^−/−^ mice (*n* = 15 cells from 5 mice per group). (I) Representative traces of sEPSCs in brain slices of SPF WT, *Scgn*^−/−^, *Scgn*^−/−^ + NaB, *Scgn*^−/−^ + Epacadostat, and WT + QA mice. Scale bars: 500 ms (horizontal), 20 pA (vertical). (J) Cumulative distribution of sEPSC amplitudes recorded in pyramidal neurons from SPF WT, *Scgn*^−/−^, *Scgn*^−/−^ + NaB, *Scgn*^−/−^ + Epacadostat, and WT + QA mice (*n* = 10 cells from 4 mice per group). (K) Mean sEPSC amplitudes in pyramidal neurons from SPF WT, *Scgn*^−/−^, *Scgn*^−/−^ + NaB, *Scgn*^−/−^ + Epacadostat, and WT + QA mice (*n* = 10 cells from 4 mice per group). (L) Cumulative distribution of inter-event intervals of sEPSCs in pyramidal neurons from SPF WT, *Scgn*^−/−^, *Scgn*^−/−^ + NaB, *Scgn*^−/−^ + Epacadostat, and WT + QA mice (*n* = 10 cells from 4 mice per group). (M) Mean sEPSC frequencies in pyramidal neurons from SPF WT, *Scgn*^−/−^, *Scgn*^−/−^ + NaB, *Scgn*^−/−^ + Epacadostat, and WT + QA mice (*n* = 10 cells from 4 mice per group). The data are expressed as mean ± SEM, with individual data points representing biological replicates. NS, not significant. Statistical analysis: One-way ANOVA followed by Tukey’s post hoc test was conducted for (B), (C), (F), (H), (K), and (M).

Collectively, these findings demonstrate that the aberrant hyperexcitability of pyramidal neurons in the mPFC contributes to anxiety-like phenotypes observed in SCGN-deficient mice, and that butyrate effectively alleviates this pathological hyperexcitability.

## Discussion

SCGN is expressed in neurosecretory cells, where it regulates hormone release. Its expression has been reported to be markedly reduced in pancreatic β cells and other endocrine-rich tissues in diabetic mice, indicating its involvement in the pathogenesis of diabetes [21]. Utilizing *Scgn*^−/−^ mice as a model, we observed that SCGN deficiency led to considerable alterations in gut microbiota composition. Behavioral assessments conducted under both SPF and GF conditions revealed pronounced anxiety-related differences, indicating that SCGN deficiency-induced microbiota changes may influence neural activity via the gut–brain axis. These findings identify SCGN as a potential molecular link connecting metabolic dysregulation, gut microbial imbalance, and the neurobehavioral manifestations associated with diabetes.

Germ-free or antibiotic treatment substantially alleviated anxiety-like behaviors in *db/db* and *Scgn*^−/−^ mice, suggesting that the gut microbiota plays a key role in the development of diabetes-associated anxiety. Increasing evidence from studies of the gut–brain axis highlights the profound impact of the gut microbiota on the CNS [38–40]. Our findings are consistent with previous reports showing that GF conditions or antibiotic interventions can ameliorate abnormal behaviors in animal models of autism, Alzheimer’s disease (AD), and depression [15,41–43]. For example, GF mice display reduced anxiety- and depression-like behaviors, antibiotic treatment improves social interactions in autism models, and GF conditions delay the onset of cognitive impairments in models of AD [15,41–43]. In our study, we identified *Odoribacter splanchnicus* as a component of the gut microbiota associated with anxiety-like behaviors. This microorganism plays a role in the production of SCFAs, including butyrate [44]. Large-scale multi-omics population studies have shown that *Odoribacter splanchnicus* may act as a “beneficial metabolic bacterium” contributing to the maintenance of CNS function and behavioral homeostasis [45]. Additionally, a systematic review of the gut microbiota in AD identifies *Odoribacter splanchnicus* as one of the dysbiotic microbiota associated with AD, further supporting its potential relevance to neurodegenerative diseases and highlighting its importance for future mechanistic investigations [46]. Oral supplementation with this bacterium ameliorated anxiety-like behaviors and decreased mPFC l-kynurenine and QA levels, even though glucose intolerance persisted. This dissociation between metabolic and behavioral phenotypes suggests that butyrate may function as a biological mediator of anxiety independent of glycemic severity. The finding is consistent with clinical observations that affective symptoms in some patients with type 2 diabetes do not correlate substantially with HbA1c levels, implying pathological mechanisms beyond glucose control [47,48]. From a therapeutic perspective, targeting gut-derived metabolites may provide an intervention avenue for diabetes-associated anxiety even when metabolic control remains suboptimal.

Lifelong GF rearing from birth selectively alleviated anxiety-like behaviors in *Scgn*^−/−^ mice; however, it failed to ameliorate autism-like deficits in social interaction and communication. These findings suggest that the absence of SCGN establishes fixed behavioral traits. These developmentally programmed circuit set points pose considerable challenges for alteration by the sole removal of microbial inputs. Conversely, anxiety-like behaviors exhibit greater plasticity and may be modulated by continuous microbiota-dependent signals that influence cortical processes, such as myelination, and ensure microglial homeostasis [49]. Furthermore, the impact of the microbiome on behaviors associated with ASD can be context-dependent; ASD donor microbiota can convey social abnormalities in GF recipients [41].

FMT from WT or *Scgn*^−/−^ donors into GF recipients reduced plasma butyrate levels, indicating that this deficit is primarily associated with gut microbiota dysbiosis. Previous studies have shown that probiotic intervention elevates SCFA levels, particularly butyrate, which in turn may mitigate anxiety and depressive behaviors induced by maternal separation stress [39,50]. Butyrate is produced by microbial fermentation of dietary fiber and can enter the systemic circulation [51]. The intriguing prospect that butyrate may cross the blood–brain barrier [52] opens new avenues for research, although the precise neuroactive mechanisms underlying its effects remain unclear. Our metabolomic profiling revealed a marked activation of the tryptophan metabolic pathway in the mPFC of *Scgn*^−/−^ mice, concomitant with elevated QA levels. Tryptophan metabolism through the KP generates bioactive metabolites, including QA, which are regulated by the rate-limiting enzyme IDO1 [53]. KP dysregulation is strongly implicated in multiple neuropsychiatric diseases [37]. Consistently, we observed substantially increased IDO1 and KYNU protein levels in the mPFC of *Scgn*^−/−^ mice, indicating enhanced KP activity. Although previous studies have shown a link between sustained IDO1 up-regulation and depression-like behaviors mediated by the prefrontal cortex [42], its role in anxiety is less well-defined. To test whether this mechanism is confined to the SCGN-deficient model, we validated it in 2 independent diabetic models: HFD-induced obese mice and *db/db* mice. Both models exhibited anxiety-like behaviors, accompanied by up-regulated mPFC IDO1/KYNU expression and elevated l-kynurenine and QA. These findings suggest that excessive activation of the tryptophan–kynurenine pathway may represent a shared neuropathological feature across different diabetic models, rather than a strain-specific artifact of SCGN deletion. A recent meta-analysis in patients with type 2 diabetes mellitus (T2DM) reported substantial elevations in peripheral kynurenine levels, together with increased downstream metabolites including QA, further supporting the activation of the tryptophan–kynurenine pathway in diabetic pathophysiology [54]. Importantly, FMT decreased QA levels in the mPFC, highlighting the regulatory role of the microbiota in host kynurenine metabolism and its therapeutic potential. Given that butyrate, a principal SCFA, can suppress IDO1 expression [36], we hypothesized that butyrate deficiency may drive KP pathway activation and contribute to the accumulation of QA in the mPFC. Consistently, butyrate supplementation alleviated anxiety-like behaviors in *Scgn*^−/−^ mice while concomitantly suppressing activity in the KP pathway.

Our in situ hybridization results revealed primary colocalization of IDO1 with neurons in the mPFC, with minimal overlap in astrocytes or microglia. While current literature indicates predominant IDO1 expression in astrocytes and microglia in the brain [44], earlier in vitro studies have reported neuronal localization [55]. This discrepancy may reflect differences in methodology. Notably, *Scgn*^−/−^ mice exhibited markedly increased θ frequency in mPFC pyramidal neurons, consistent with the established association between heightened θ activity and anxiety behaviors in the literature [56]. However, butyrate supplementation normalized this aberrant θ activity, supporting its regulatory potential. This modulation is likely facilitated through regulating the IDO1-mediated KP.

QA is an NMDA receptor agonist that, at elevated levels, can promote excessive neuronal excitotoxicity via receptor overactivation, potentially leading to oxidative stress and apoptosis [57–59]. In this study, bilateral mPFC injection of QA directly induced anxiety-like behaviors in WT mice, demonstrating that QA-driven behavioral effects are independent of systemic metabolic deterioration. In acute ex vivo slice experiments, QA treatment increased the frequency of sEPSCs in WT neurons, phenocopying the hyperexcitable state observed in SCGN-deficient neurons; conversely, sodium butyrate or the IDO1 inhibitor epacadostat normalized the aberrant firing in knockout slices. The dissociation between increased frequency and unchanged amplitude indicates a presynaptic mechanism—most likely an elevated probability of glutamate release rather than altered postsynaptic receptor sensitivity [60,61]. This mechanism can disrupt information processing within the mPFC circuits and influence downstream limbic regions, such as the amygdala, thereby hindering emotional regulation and contributing to the manifestation of anxiety. Our findings align with recent studies [62–67], which confirm that the stress-induced hyperexcitability of pyramidal neurons represents a fundamental pathological mechanism involved in anxiety disorders.

SCFAs, particularly butyrate, have attracted increasing attention for their beneficial effects on gastrointestinal and neuropsychiatric health [52]. Butyrate primarily ameliorates anxiety and related mental health conditions by modulating gut microbiota composition and regulating inflammatory pathways, thereby influencing communication along the gut–brain axis [68–70]. A randomized controlled trial in patients with ulcerative colitis reported that butyrate salt supplementation reduces disease severity and alleviates comorbid psychological symptoms, including anxiety and depression [71]. Our study further demonstrated that oral administration of butyrate effectively ameliorated anxiety-like behaviors in SCGN-deficient diabetic mice, underscoring its therapeutic potential for diabetes-associated anxiety. Nevertheless, our clinical data showing reduced butyrate levels in diabetic patients with anxiety, together with the preclinical findings, highlight the therapeutic potential of butyrate supplementation; clinical studies are still required to determine whether butyrate can provide meaningful benefit in these patients, either alone or in combination with existing anxiolytic therapies. Furthermore, the strategic modulation of the gut microbiota and their metabolites may represent a promising avenue for developing novel neuropsychiatric interventions, expanding the therapeutic landscape for anxiety disorders.

This study has several limitations that should be acknowledged. First, using a global *Scgn* knockout model precludes definitive attribution of the observed phenotypes to specific cell types or tissues and cannot rule out potential developmental effects. Therefore, tissue- or cell type-specific deletion or rescue studies will be necessary to determine the cellular origin of these effects. Second, all experiments were conducted exclusively in male mice, and whether the identified gut–brain axis operates similarly in females remains to be determined. Sex-dependent differences in anxiety-like behaviors have been documented, with anxiety indices varying across estrous stages. For example, diestrus females have been reported to exhibit higher anxiety indices than proestrus females or males in the open-field, light–dark box, and elevated plus maze tests [72]. Therefore, further studies incorporating both sexes and appropriate control of estrous-cycle status will be important for determining potential sex dependence of the gut–brain mechanisms identified here. Third, direct validation in human samples was limited. In particular, due to constraints on sample acquisition, we cannot assess tissue expression of SCGN/IDO1/KYNU in diabetic versus nondiabetic subjects. Fourth, although our FMT experiments support a microbiota-mediated mechanism, potential cage effects on microbial composition and transplantation outcomes cannot be completely excluded. Fifth, although our findings implicate butyrate in the regulation of the IDO1–KYNU–QA pathway, the specific cellular and molecular targets through which butyrate exerts its effects remain to be determined. Finally, our microbiome analysis did not incorporate absolute microbial abundance normalization using spike-in controls or direct quantification of genes involved in the butyrate biosynthesis.

In conclusion, our findings support a pathogenic model in which SCGN deficiency is associated with gut microbiota dysbiosis and reduced butyrate production, accompanied by excessive activation of tryptophan metabolism in the mPFC. These changes lead to QA accumulation and subsequent hyperexcitability of pyramidal neurons, ultimately contributing to anxiety-like behaviors. These insights into the mechanisms of action highlight the potential of butyrate supplementation as a promising therapeutic strategy for addressing anxiety-like behaviors associated with diabetes. Moreover, within the context of precision medicine, a deeper understanding of this mechanism may facilitate the development of targeted therapies, thereby broadening treatment options for diabetes-associated anxiety and fostering further research into the underlying mechanisms.

## Materials and Methods

### Animals

All experiments were conducted in accordance with the established guidelines for the Care and Use of Laboratory Animals and received formal approval by the Ethics Committee of the Experimental Animal Center at the Army Medical University (AMUWEC20237517).

C57BL/6JGpt-Scgn^em1Cd15791^/Gpt (*Scgn*^−/−^) mice were acquired from GemPharmatech, while C57BL/6J WT mice were purchased from Charles River Laboratories. Moreover, within the context, GF derivatives for both strains were produced by Mingqian Microbe Ltd. The GF WT and *Scgn*^−/−^ mice were generated and maintained at Kunshan Mingqian Microbiology Research Institute Co. Ltd. (Kunshan, Jiangsu, China; ID: RB8101-240608), using flexible-film GF isolators supplied by Class Biologically Clean Ltd. (Madison, WI, USA). The gnotobiotic platform at Kunshan Mingqian has been validated through peer-reviewed publications [73].

*db/db* (BKS.Cg-Dock7^m^ +/+ Lepr^db^) and *db/m* mice were obtained from Cavens Model Animal Co. Ltd. These mice received an antibiotic cocktail in their drinking water, comprising neomycin (1 g/l), metronidazole (1 g/l), ampicillin (1 g/l), and vancomycin (500 mg/l) for 1 week. The solution was replaced every 2 d, in accordance with established protocols [74–76]. Detailed timelines for specific treatments relative to other interventions are outlined in the respective figure legends. For the groups receiving butyrate treatment, sodium butyrate (5 g/l) was administered in the drinking water for 7 d [77–79].

For FMT, fresh fecal pellets were collected from SPF WT and *Scgn*^−/−^ mice at 84 d of age, suspended in phosphate-buffered saline (PBS) at 5% (w/v), homogenized, and filtered through a sterile 800-mesh screen. *Odoribacter splanchnicus* (strain DSM 20712, 1 × 10^9^ CFU/ml) was obtained from the German Collection of Microorganisms and Cell Cultures (DSMZ, Germany). Bacterial identity was verified by 16S rRNA PCR using the following primers: forward, 5‘-ATGTAATGATGAGCACTCTAACGG-3‘; reverse, 5‘-GGCTTTTGAGATTGGCATCC-3‘. Recipients received 200 μl of this suspension via oral gavage 3 times per week [80–82]. The FMT suspensions were utilized within 1 d of preparation. Antibiotic and butyrate drinking solutions were prepared prior to administration and were replaced every 2 d, while standard drinking water was provided at all other times. Cage changes were conducted twice a week under GF conditions.

For behavioral assays, male mice were utilized exclusively and housed in groups of 3 to 5 per cage, under a standardized 12-h light/dark cycle (07:00 to 19:00 light phase), with ad libitum access to food and water unless otherwise specified. The ambient temperature was maintained between 23 and 25 °C, and humidity was controlled at 55% to 62%, with all conditions automatically regulated.

The term “*db/db*” refers to Leprdb/Leprdb mice, which are homozygous for a spontaneous recessive mutation in the leptin receptor gene (Lepr, also known as Ob-R) on a C57BL/KsJ background and serve as a model for T2DM. “Lep” denotes the leptin gene. DM and T2D refer to diabetes mellitus and type 2 diabetes mellitus, respectively.

Animals were randomly allocated to experimental groups using a computer-generated random number sequence (R software, sample function). Samples and tissue sections were identified by codes, and investigators performing data acquisition and analysis (behavioral video analysis, densitometric quantification, patch-clamp recordings, and IF cell counting) were blinded to genotype and treatment group.

### Human participants

We recruited 51 patients with T2DM from the Department of Endocrinology, First Affiliated Hospital of Army Medical University, PLA. Inclusion criteria were as follows: diagnosis of T2DM, 18 to 75 years, any sex, and willingness to provide written informed consent. Exclusion criteria were as follows: a history of neurological or psychiatric disorders; use of psychotropic medications within the preceding month; a family history of psychiatric disorders; a history of intestinal diseases; the presence of acute diabetic complications; and use of antibiotics, probiotics, or anxiolytic medications within the preceding 4 weeks. All participants provided written informed consent prior to enrollment. The study protocol was approved by the Ethics Committee of the First Affiliated Hospital of Army Medical University [No. (A) KY2026162] and conducted in accordance with the Declaration of Helsinki.

### FISH and IF staining

As previously described [83], mice were transcardially perfused with PBS, and brain tissues were subsequently fixed in 4% paraformaldehyde at 4 °C for 48 h. The tissues were then paraffin-embedded and sectioned coronally into 5-μm thickness using a Leica microtome. For fluorescence in situ hybridization (FISH) experiments, the sections were mounted on Sevier Swe precoated slides and air-dried. Multicolor FISH experiments were performed according to the manufacturer’s specifications (Sevier Fluorescent Multiplex Assay kit) using a custom-designed SweAMI ISH probe (Sevier; NM_008324.1).

For IF experiments, paraffin sections were blocked overnight using a solution containing 1× PBS, 5% bovine plasma albumin, and 0.1% Triton X-100. Primary antibodies, diluted in the blocking solution, were applied for 24 h at 4 °C: rabbit anti-c-Fos (1:2,000; Synaptic Systems, 226003), rabbit anti-GFAP (1:500; Proteintech, 16825-1-AP), mouse anti-NEUN (1:500; Proteintech, 26975-1-AP), and rabbit anti-IBA1 (1:1,000; Wako, 016-26721). Following 3 washes with PBS, the sections were incubated with Alexa Fluor-conjugated secondary antibodies (1:500; Thermo Fisher Scientific, A11039, A21206, and A10042) for 2 h at room temperature. For c-Fos immunostaining, brain samples were collected 90 min following the elevated plus maze test to capture c-Fos expression induced by behavioral stimulation [84–86]. The slides were mounted using a fluorescent mounting medium and imaged using a Zeiss LSM880 confocal microscope.

### Stereotaxic surgery and electrode implantation

As previously described [87], mice were anesthetized by isoflurane inhalation (1.5% in oxygen at a flow rate of 0.8 l/min) and then secured in a small-animal stereotaxic instrument (RWD 68801). Following proper placement, a 32-channel tetrode electrode (Kedou BC) was implanted in the mPFC using the following coordinates: anterior–posterior (AP): +1.94 mm, medial–lateral (ML): +0.45 mm, and dorsal–ventral (DV): −2.4 mm. The electrodes were secured with dental cement, and the mice were subsequently placed in a cage for a 5-d postoperative recovery before proceeding with further experimental procedures.

### In vivo electrophysiology

As previously described [88], the extracellular electrical signals from implanted tetrodes were initially amplified via a headstage (CerePlex μ, 32 channels, Blackrock Ltd). Following stereotaxic electrode implantation, the mice were individually housed in ventilated SPF cages with nesting material and shelters to prevent damage to the externalized implants. The single housing was maintained consistently throughout the behavioral testing period, which lasted up to 4 weeks, and was applied uniformly across all groups. The animals received postoperative analgesia and were given at least 7 d to recover before testing commenced [89]. Output signals were continuously digitized at 30 kHz with 16-bit resolution using the Blackrock cereplex direct system (Blackrock Ltd.). Signals were down-sampled to 30 kHz. Spike waveforms from individual neurons were detected using Kilosort automatic clustering software (https://github.com/MouseLand/Kilosort). The units were manually curated using the Phy software (https://github.com/cortex-lab/phy) employing rigorous validation criteria: waveform shapes, amplitude modulation across tetrode channels, temporal stability, autocorrelation (to assess the refractory period of single units), and cross-correlation (to evaluate shared refractory periods within single units). Putative pyramidal neurons and putative interneurons were classified based on average spike width and spike shape. The peak widths of the recorded neurons showed a bimodal distribution, which was subsequently categorized using hierarchical clustering in Matlab [89]. Spike counts were quantified using NeuroExplorer, and firing rates were calculated in 0.5-s intervals over 3 s before and after entry into the open arms. Neurons exhibiting a greater than 100-fold increase in firing rate after entry were classified as anxiety pyramidal neurons and anxiety interneurons. The LFP data were recorded at a sampling frequency of 1,000 Hz using a headstage. A low-pass filter was applied to isolate extracellular potentials in the range of 0.5 to 251 Hz. For the LFP analysis, NeuroExplorer software was used to compute the power spectrum. Following this, *Z*-score normalization was performed, and the average PSD was calculated across standard frequency bands: delta waves (0.5 to 4 Hz), theta waves (4 to 8 Hz), alpha waves (8 to 13 Hz), beta waves (13 to 30 Hz), and gamma waves (30 to 100 Hz).

### In vitro electrophysiology

As previously described [90],experimental mice were subjected to deep anesthesia induced by intraperitoneal injection of Avertin (Dowobio, DW3101, 20 μl/g) and then underwent rapid decapitation. The brain tissues were immediately extracted, and 300-μm-thick coronal slices containing the mPFC were prepared using a vibratome (Instruments, Campden Model 5100mz-86) while fully submerged in ice-cold, oxygenated (95% O_2_ and 5% CO_2_) slicing artificial cerebrospinal fluid (slicing ACSF; pH 7.4) with the following composition: 93 mM N-methyl-D-glucamine (NMDG), 93 mM HCl, 2.5 mM KCl, 10 mM MgSO_4_, 1.2 mM NaH_2_PO_4_, 30 mM NaHCO_3_, 25 mM glucose, 20 mM Hepes, 5 mM sodium ascorbate, 3 mM sodium pyruvate, 2 mM thiourea, and 0.5 mM CaCl_2_. Slices underwent sequential recovery: 30 min in oxygenated standard ACSF at 35 °C, followed by 30 min at room temperature, then 1-h equilibration in oxygenated (95% O_2_ and 5% CO_2_) ACSF (pH adjusted to 7.4). The ACSF composition was as follows: 126 mM NaCl, 2.5 mM KCl, 1.25 mM NaH_2_PO_4_, 10 mM glucose, 25 mM NaHCO_3_, and 2 mM CaCl_2_. Slices from each animal were randomly assigned to pharmacological treatment conditions using a pre-generated randomization list. Whole-cell recordings were performed with patch pipettes filled with intracellular solution (pH 7.4): 133 mM potassium gluconate, 18 mM NaCl, 0.6 mM EGTA, 4 mM MgATP, 5 mM phosphocreatine, 10 mM Hepes, 0.3 mM NaGTP. Membrane potential was clamped at −70 mV. sEPSCs were recorded in ACSF containing 5 μM picrotoxin (PTX) at a ratio of 1:100 (PTX:ACSF); sIPSCs were recorded with 2 μM 6-cyano-7-nitroquinoxaline-2,3-dione (CNQX) and 5 μM D-2-amino-5-phosphonovalericacid (D-AP5) at a 1:1:100 ratio (CNQX:D-AP5:ACSF). Signals were amplified using a Multiclamp 700B amplifier (Molecular Devices), and data were acquired and analyzed with Clampfit 10.0 software (Molecular Devices).

### Behavioral assays

As previously described [91], the experimental mice were acclimated in the experimental apparatus for 30 min 1 d prior to behavioral assessment. All behavioral tests were conducted during the light phase, with a minimum of 24 h between tests. Experimental arenas were meticulously sanitized with 75% ethanol between trials to minimize olfactory cues. All behavioral data were analyzed using EthoVision 11.0 software (Noldus) and reviewed by 3 trained observers who were blinded to the treatments.

#### Open-field test

As previously described [92], locomotor activity and anxiety-like behavior were assessed in a 40 cm × 40 cm × 30 cm open field. The arena was digitally divided into central and peripheral areas using EthoVision 11.0 software. Mice were placed in the central zone, and their trajectories were recorded for 30 min. Latency to enter the periphery and area-specific locomotion were recorded and analyzed.

#### Elevated plus maze test

Anxiety-related behavior was evaluated in an elevated plus maze, consisting of 2 open (30 cm × 5 cm) and 2 enclosed (30 cm × 5 cm × 15 cm) arms arranged in a plus shape [93]. Mice were positioned at the central junction facing an open arm, and their movements were recorded for 10 min using EthoVision 11.0 software. The time spent in open versus closed arms and the total number of entries into the open arms were recorded and analyzed to evaluate anxiety levels.

#### Light–dark box test

Anxiety-like responses were measured using a 2-chamber apparatus with light and dark compartment [94]. At the onset of the test, mice were placed in the light chamber. Their movements between the compartments were recorded for 10 min using EthoVision 11.0 software. The analysis included time spent in each compartment and the number of transitions between them to assess anxiety levels.

#### Self-grooming test

The self-grooming test was performed as previously described [95]. Briefly, mice were individually placed in a standard transparent testing cage and allowed to acclimate. Each session lasted 20 min and was recorded with a high-definition video camera. The initial 10 min served as an adaptation phase. The duration of self-grooming behavior was measured during the final 10 min of the session by an experienced observer who remained blinded to experimental group assignments.

#### Marble burying test

The marble burying test was conducted as described by Liu et al. [96]. Mice were placed individually in standard test cages (27 cm × 16.5 cm × 12.5 cm) filled with 6 to 7 cm of corn cob bedding. Twenty black glass marbles (diameter: 1.5 cm) were systematically arranged in a 4 × 5 grid on the bedding surface. The mice were allowed to explore the cage freely for 30 min under dim light conditions (~15 lx). At the end of the test, the number of marbles that had been buried to a depth of at least three-quarters in the bedding was counted.

#### Three-chamber social interaction test

As previously described [97], the 3-chamber social interaction test was performed in a clear acrylic box measuring 60 cm × 40 cm × 22 cm, divided into 3 equal compartments, with minor modifications. During the habituation phase, each mouse was allowed to explore all 3 empty chambers for 10 min freely. In the sociability phase, an unfamiliar age- and sex-matched mouse [stranger 1 (S1)] was placed in a wire cage in one side chamber. In contrast, an identical empty wire cage [object (O)] was positioned in the opposite side chamber. The test mouse was reintroduced into the arena and allowed to explore for an additional 10 min. After a 5-min interval, the test mouse was briefly removed, and a new, unfamiliar mouse [stranger 2 (S2)] was placed in the previously empty wire cage. In contrast, the original stranger mouse (S1) remained in place. The test mouse was then given another 10 min to explore all 3 chambers, allowing assessment of preference for social novelty. All sessions were recorded and analyzed using EthoVision software. The time spent sniffing each wire cage was measured, and the preference index for social novelty was calculated as the difference between S2 and S1 during the social novelty phase.

#### Forced swim test

To assess depressive-like behavior, transparent cylinders filled with water at approximately 25 °C were employed [98]. The water depth was sufficient to prevent direct contact with the bottom. During the typical 5-min test, we quantified immobility time, defined as passive floating with minimal movements to keep the head above water, versus active swimming/struggling time, characterized by vigorous paw movements, wall scraping, or diving. Data were analyzed using EthoVision 11.0 software.

#### Tail suspension test

Mice were suspended using adhesive tape affixed 2 cm from the tip of their tails, preventing contact with surfaces and promoting active struggling behaviors [99]. During standardized 6-min trials, we measured the duration of immobility, defined as the absence of escape-oriented movements except for respiration. The initial 2 min served as an acclimation period, while immobility analysis focused on the final 4 min using EthoVision 11.0 software.

### Microbiome

As previously described [100], samples were collected using sterile techniques and stored at −80 °C. DNA extraction was performed in accordance with the manufacturer’s instructions using the E.Z.N.A. Soil DNA Kit (Omega Bio-tek, Norcross, GA). The concentration and purity of the extracted DNA were assessed using the Quantus Fluorometer (Promega) and NanoDrop2000 (Thermo Fisher Scientific), and quality was verified by 1% agarose gel electrophoresis. For paired-end library construction, the DNA was fragmented to an average size of 350 base pairs using the Covaris M220 (Gene Company, China). The library was constructed using the NEXTflex Rapid DNA-Seq Kit (Bioo Scientific). Subsequently, the library was enriched by PCR amplification and purified using magnetic beads. Sequencing was performed on the Illumina NovaSeq PE250 platform, and the raw sequencing data were deposited in the NCBI Sequence Read Archive (SRA) under accession number PRJNA1391800. α-Diversity analysis was performed using the Shannon, Chao, Ace, and Simpson indices. PCA was conducted to analyze microbial structure. The LEfSe method was implemented to identify differentially enriched bacterial taxa across treatment groups. Finally, Spearman correlation analysis was conducted using the Wekemo Bioincloud platform to examine the relationships between gut microbiota composition and host parameters.

### Metabolomics

For liquid chromatography (LC)-MS samples [101], tissue specimens were flash-frozen in liquid nitrogen to prevent metabolite degradation. After drying, the samples were stored at −80 °C until analysis. Following grinding, metabolites were extracted using a biphasic solvent system (methanol:chloroform:water, 2:2:1, v/v/v) to separate polar and nonpolar metabolites effectively.

Metabolomic analysis was performed using a high-resolution LC-MS/MS system from Agilent Technologies, with samples examined in both positive and negative ion modes. Sample preparation, instrument operation, and detection were performed by Gene Denovo Biotechnology Co. Ltd. (Guangzhou, China). Subsequent analysis steps were performed on the Gene Denovo platform (https://www.omicshare.com/), as briefly described below. PCA and orthogonal projection to latent structures–discriminant analysis (OPLS-DA) were used to evaluate sample clustering and to identify marked metabolites. Differentially expressed metabolites were selected based on VIP scores (VIP > 1) and false discovery rate-adjusted *P* values (FDR < 0.05). Data visualization was conducted using R software. Moreover, MSEA was performed to identify biologically relevant perturbed pathways, and global testing of quantitative enrichment analysis (QEA) was conducted using the R package MSEAp.

Fifty-one patients with T2DM were enrolled in the study. After an overnight fast, peripheral venous blood was collected into EDTA-anticoagulated tubes and centrifuged at 3,000*g* for 10 min at 4 °C to obtain plasma, which was immediately aliquoted and stored at −80 °C until analysis. Fasting plasma butyrate concentrations were quantified by GC-MS using a standard calibration curve.

For GC/MS samples [102], after tissue collection, 50 μl of 30% phosphoric acid solution was added, along with 300 μl of acetone. The mixture was then homogenized for 3 min at 12,000 rpm and subsequently centrifuged for 10 min. The supernatant was carefully collected and diluted 10-fold prior to instrumental analysis. Data acquisition was performed using an Agilent gas chromatography system (Agilent Technologies; Agilent 7820), while data processing and quantification were performed utilizing Quant My Way software (Agilent Technologies).

The data for this analysis are available in the MetaboLights website (MTBLS13477).

### Western blot experiment

The Western blot analysis was performed according to standard methods [103]. Briefly, proteins were extracted and quantified from the mPFC using the bicinchoninic acid (BCA) method (Beyotime, P0012). The samples were subjected to electrophoresis using ExpressPlus PAGE (Genscript, M01115C) and subsequently transferred to polyvinylidene difluoride membranes. After blocking, the membranes were incubated overnight at 4 °C with primary antibodies. The primary antibodies utilized in this study included the following: anti-β-actin (1:1,000; Beyotime, AF0003), anti-KYNU (1:1,000; Proteintech, 11796-1-AP), anti-IDO1 (1:1,000; Abmart, MU131118M), anti-KMO (1:1,000; Proteintech, 10698-1-AP), anti-ACSMD (1:1,000; Invitrogen, PA5-21790), and anti-HAAO (1:1,000; Abcam, ab113716). Following this, the membranes were washed and incubated with the respective secondary antibodies (1:5,000; Abclonal, AS003/AS014/AS028) for 2 h at room temperature. The membranes were washed again and finally visualized using the Super ECL detection reagent kit (Yeasen, 36208ES60).

### Statistical analysis

All statistical analyses were conducted using GraphPad Prism (version 9) software. Mice were randomly assigned to experimental groups, with randomization implemented where feasible during data collection. Data collection and analysis were not blinded to experimental conditions. All behavioral experiments employed automated computer systems to ensure unbiased data acquisition and analysis. Multiple comparisons were analyzed by one-way analysis of variance (ANOVA) followed by Tukey’s post hoc test. Unpaired and paired *t* tests were employed for intergroup and intragroup comparisons, respectively. Statistical methods did not predetermine sample sizes, nor were group assignments randomized statistically. Detailed statistical approaches were specified in the respective figure legends. All data are presented as mean ± SEM (α = 0.05). NS, not significant.

## Ethical Approval

All animal experiments were conducted in accordance with the established guidelines for the Care and Use of Laboratory Animals and were approved by the Ethics Committee of the Experimental Animal Center at the Army Medical University (AMUWEC20237517). For the human study, all participants provided written informed consent prior to enrollment, and the study protocol was approved by the Ethics Committee of the First Affiliated Hospital of Army Medical University [No. (A) KY2026162] and conducted in accordance with the Declaration of Helsinki.

## Data Availability

The sequencing data and metabolomics data reported in this paper have been deposited in the NCBI Sequence Read Archive (PRJNA1391800) and MetaboLights (MTBLS13477), respectively, and are publicly available. All other data supporting the findings of this study are available from the corresponding authors upon reasonable request.
