# Supplementary material for: Butyrate Deficiency due to Microbial Dysbiosis Promotes Anxiety
Source: Research (Wash D C). 2026 Nov 9;9:1470. doi: 10.34133/research.1470 (PMC13745766; doi:10.34133/research.1470)
Supplement: Supplementary file 1 [file research.1470.f1.zip › sf1.pdf]

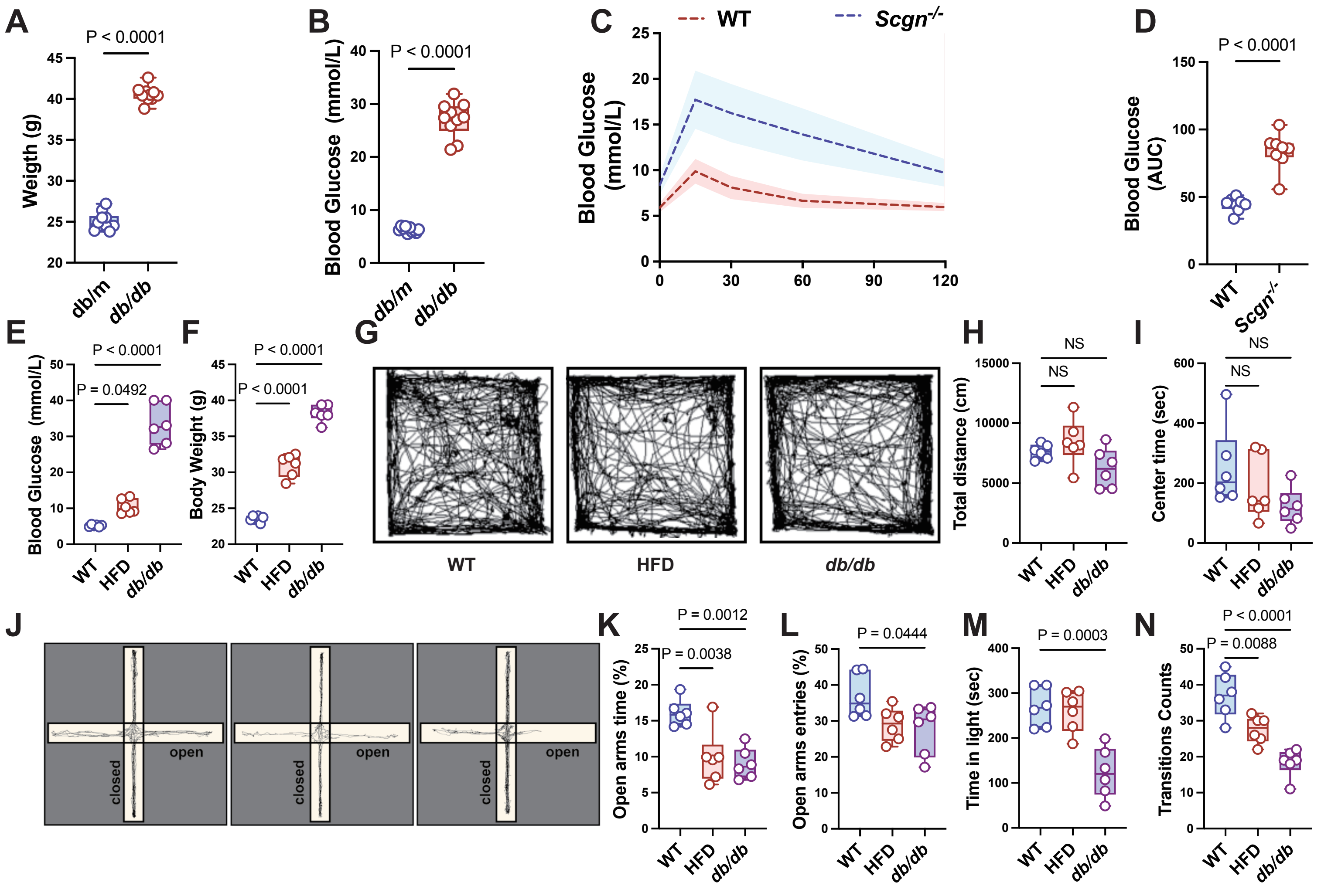
A
P < 0.0001
Weigth (g)
db/m
db/db
B
P < 0.0001
Blood Glucose (mmol/L)
db/m
db/db
C
WT
Scgn-/-
Blood Glucose (mmol/L)
0
30
60
90
120
D
P < 0.0001
Blood Glucose (AUC)
WT
Scgn-/-
E
P < 0.0001
P = 0.0492
Blood Glucose (mmol/L)
WT
HFD
db/db
F
P < 0.0001
P < 0.0001
Body Weight (g)
WT
HFD
db/db
G
WT
HFD
db/db
H
NS
NS
Total distance (cm)
WT
HFD
db/db
I
NS
NS
Center time (sec)
WT
HFD
db/db
J
closed
open
closed
open
closed
open
K
P = 0.0012
P = 0.0038
Open arms time (%)
WT
HFD
db/db
L
P = 0.0444
Open arms entries (%)
WT
HFD
db/db
M
P = 0.0003
Time in light (sec)
WT
HFD
db/db
N
P < 0.0001
P = 0.0088
Transitions Counts
WT
HFD
db/db
