# Supplementary material for: Butyrate Deficiency due to Microbial Dysbiosis Promotes Anxiety
Source: Research (Wash D C). 2026 Nov 9;9:1470. doi: 10.34133/research.1470 (PMC13745766; doi:10.34133/research.1470)
Supplement: Supplementary file 1 [file research.1470.f1.zip › sf2.pdf]

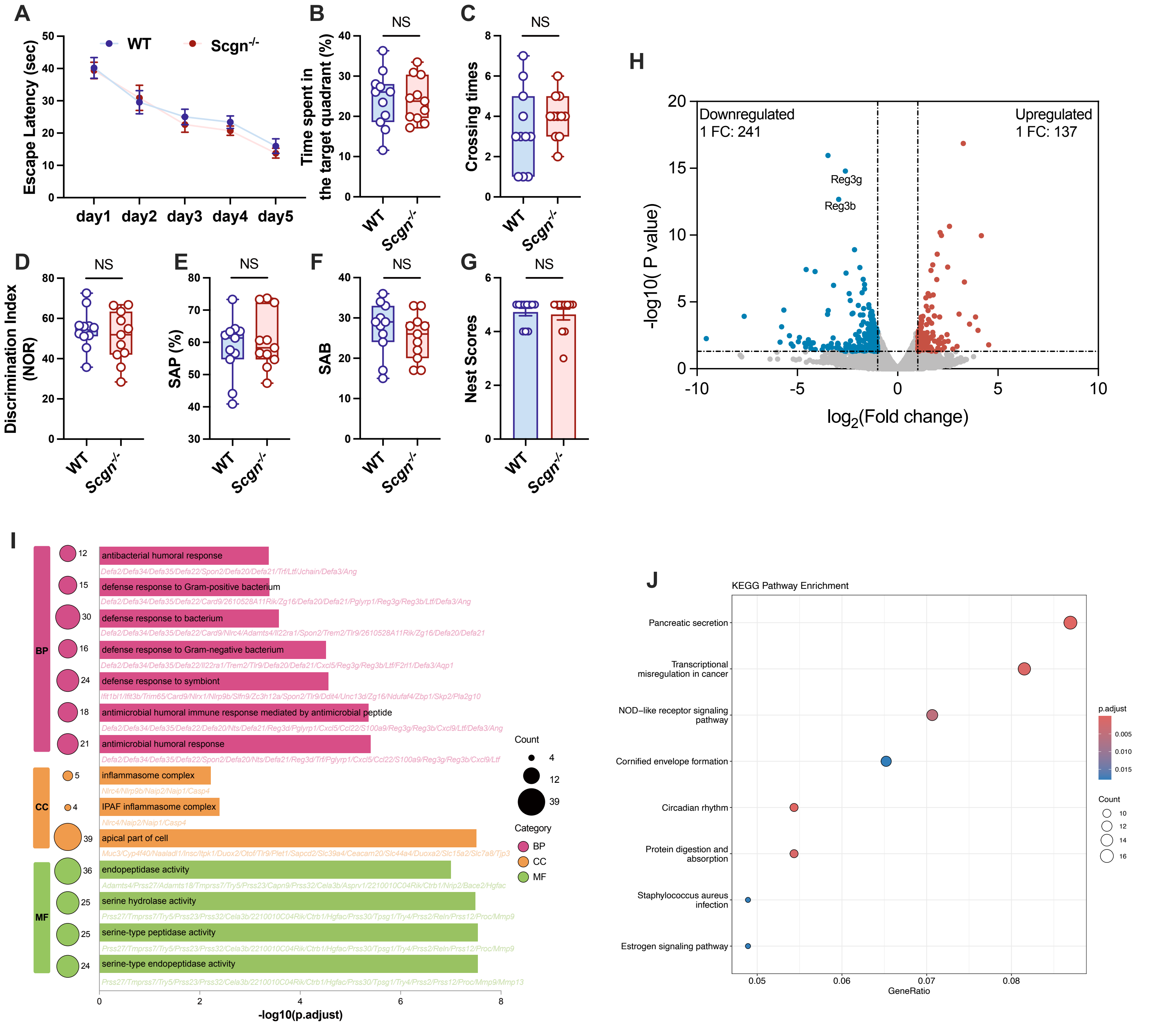
A
Escape Latency (sec)
WT
Scgn-/-
day1 day2 day3 day4 day5
B
NS
Time spent in the target quadrant (%)
WT
Scgn-/-
C
NS
Crossing times
D
NS
Discrimination Index (NOR)
E
NS
SAP (%)
F
NS
SAB
G
NS
Nest Scores
H
Downregulated
1 FC: 241
Upregulated
1 FC: 137
Reg3g
Reg3b
-log10(P value)
log2(Fold change)
I
BP
CC
MF
antibacterial humoral response
Defa2/Defa34/Defa35/Defa22/Spon2/Defa20/Defa21/Trf/Ltf/Jchain/Defa3/Ang
defense response to Gram-positive bacterium
Defa2/Defa34/Defa35/Defa22/Card9/2610528A11Rik/Zg16/Defa20/Defa21/Pglyrp1/Reg3g/Reg3b/Ltf/Defa3/Ang
defense response to bacterium
Defa2/Defa34/Defa35/Defa22/Card9/Nlrc4/Adamts4/Il22ra1/Spon2/Trem2/Tlr9/2610528A11Rik/Zg16/Defa20/Defa21
defense response to Gram-negative bacterium
Defa2/Defa34/Defa35/Defa22/Il22ra1/Trem2/Tlr9/Defa20/Defa21/Cxcl5/Reg3g/Reg3b/Ltf/F2rl1/Defa3/Aqp1
defense response to symbiont
Ifit1bl1/Ifit3b/Trim65/Card9/Nlrx1/Nlrp9b/Slfn9/Zc3h12a/Spon2/Tlr9/Ddit4/Unc13d/Zg16/Ndufaf4/Zbp1/Skp2/Pla2g10
antimicrobial humoral immune response mediated by antimicrobial peptide
Defa2/Defa34/Defa35/Defa22/Defa20/Nts/Defa21/Reg3d/Pglyrp1/Cxcl5/Ccl22/S100a9/Reg3g/Reg3b/Cxcl9/Ltf/Defa3/Ang
antimicrobial humoral response
Defa2/Defa34/Defa35/Defa22/Spon2/Defa20/Nts/Defa21/Reg3d/Trf/Pglyrp1/Cxcl5/Ccl22/S100a9/Reg3g/Reg3b/Cxcl9/Ltf
inflammasome complex
Nlrc4/Nlrp9b/Naip2/Naip1/Casp4
IPAF inflammasome complex
Nlrc4/Naip2/Naip1/Casp4
apical part of cell
Muc3/Cyp4f40/Naaladl1/Insc/Itpk1/Duox2/Otof/Tlr9/Plet1/Sapcd2/Slc39a4/Ceacam20/Slc44a4/Duoxa2/Slc15a2/Slc7a8/Tjp3
endopeptidase activity
Adamts4/Prss27/Adamts18/Tmprss7/Try5/Prss23/Capn9/Prss32/Cela3b/Asprv1/2210010C04Rik/Ctrb1/Nrip2/Bace2/Hgfac
serine hydrolase activity
Prss27/Tmprss7/Try5/Prss23/Prss32/Cela3b/2210010C04Rik/Ctrb1/Hgfac/Prss30/Tpsg1/Try4/Prss2/Reln/Prss12/Proc/Mmp9
serine-type peptidase activity
Prss27/Tmprss7/Try5/Prss23/Prss32/Cela3b/2210010C04Rik/Ctrb1/Hgfac/Prss30/Tpsg1/Try4/Prss2/Reln/Prss12/Proc/Mmp9
serine-type endopeptidase activity
Prss27/Tmprss7/Try5/Prss23/Prss32/Cela3b/2210010C04Rik/Ctrb1/Hgfac/Prss30/Tpsg1/Try4/Prss2/Prss12/Proc/Mmp9/Mmp13
Count
Category
-log10(p.adjust)
J
KEGG Pathway Enrichment
Pancreatic secretion
Transcriptional misregulation in cancer
NOD−like receptor signaling pathway
Cornified envelope formation
Circadian rhythm
Protein digestion and absorption
Staphylococcus aureus infection
Estrogen signaling pathway
GeneRatio
p.adjust
Count
