# Supplementary material for: Butyrate Deficiency due to Microbial Dysbiosis Promotes Anxiety
Source: Research (Wash D C). 2026 Nov 9;9:1470. doi: 10.34133/research.1470 (PMC13745766; doi:10.34133/research.1470)
Supplement: Supplementary file 1 [file research.1470.f1.zip › sf3.pdf]

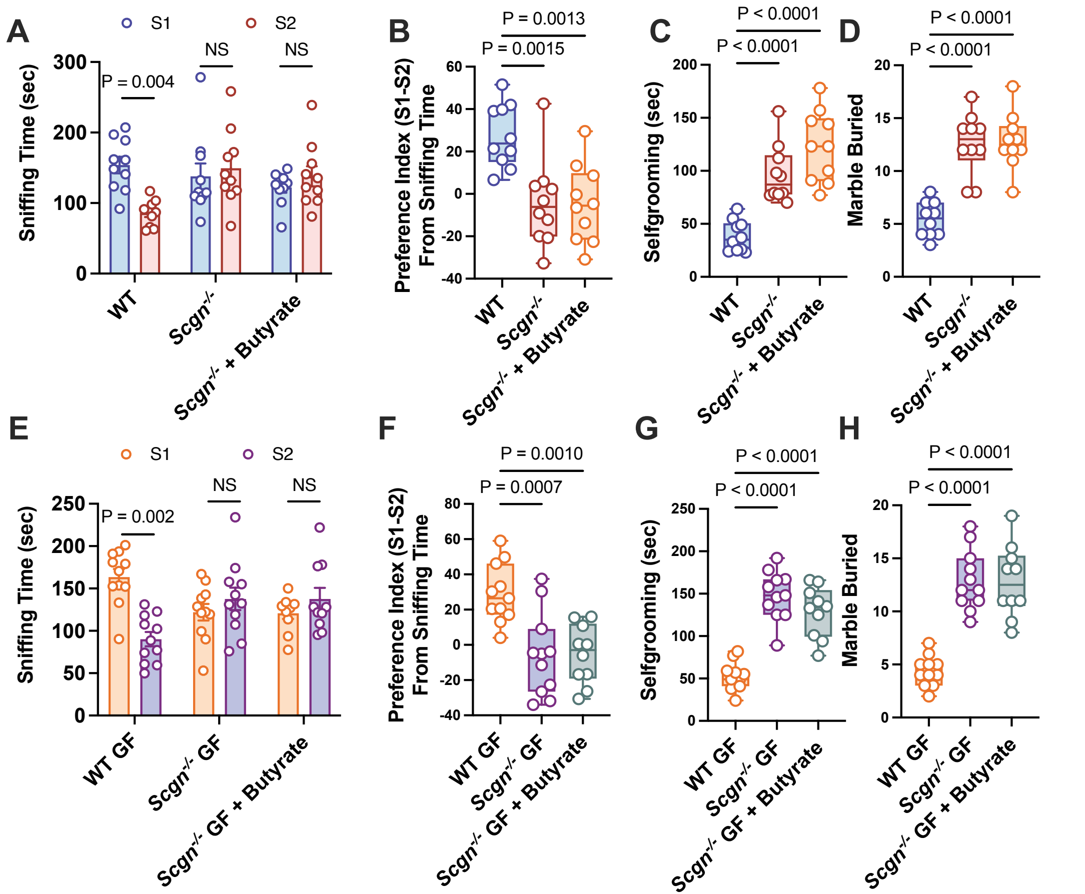

A
S1
S2
Sniffing Time (sec)
P = 0.004
NS
NS
WT
Scgn-/-
Scgn-/- + Butyrate
B
P = 0.0013
P = 0.0015
Preference Index (S1-S2) From Sniffing Time
WT
Scgn-/-
Scgn-/- + Butyrate
C
P < 0.0001
P < 0.0001
Selfgrooming (sec)
WT
Scgn-/-
Scgn-/- + Butyrate
D
P < 0.0001
P < 0.0001
Marble Buried
WT
Scgn-/-
Scgn-/- + Butyrate
E
S1
S2
Sniffing Time (sec)
P = 0.002
NS
NS
WT GF
Scgn-/- GF
Scgn-/- GF + Butyrate
F
P = 0.0010
P = 0.0007
Preference Index (S1-S2) From Sniffing Time
WT GF
Scgn-/- GF
Scgn-/- GF + Butyrate
G
P < 0.0001
P < 0.0001
Selfgrooming (sec)
WT GF
Scgn-/- GF
Scgn-/- GF + Butyrate
H
P < 0.0001
P < 0.0001
Marble Buried
WT GF
Scgn-/- GF
Scgn-/- GF + Butyrate
