# Supplementary material for: Butyrate Deficiency due to Microbial Dysbiosis Promotes Anxiety
Source: Research (Wash D C). 2026 Nov 9;9:1470. doi: 10.34133/research.1470 (PMC13745766; doi:10.34133/research.1470)
Supplement: Supplementary file 1 [file research.1470.f1.zip › sf4.pdf]

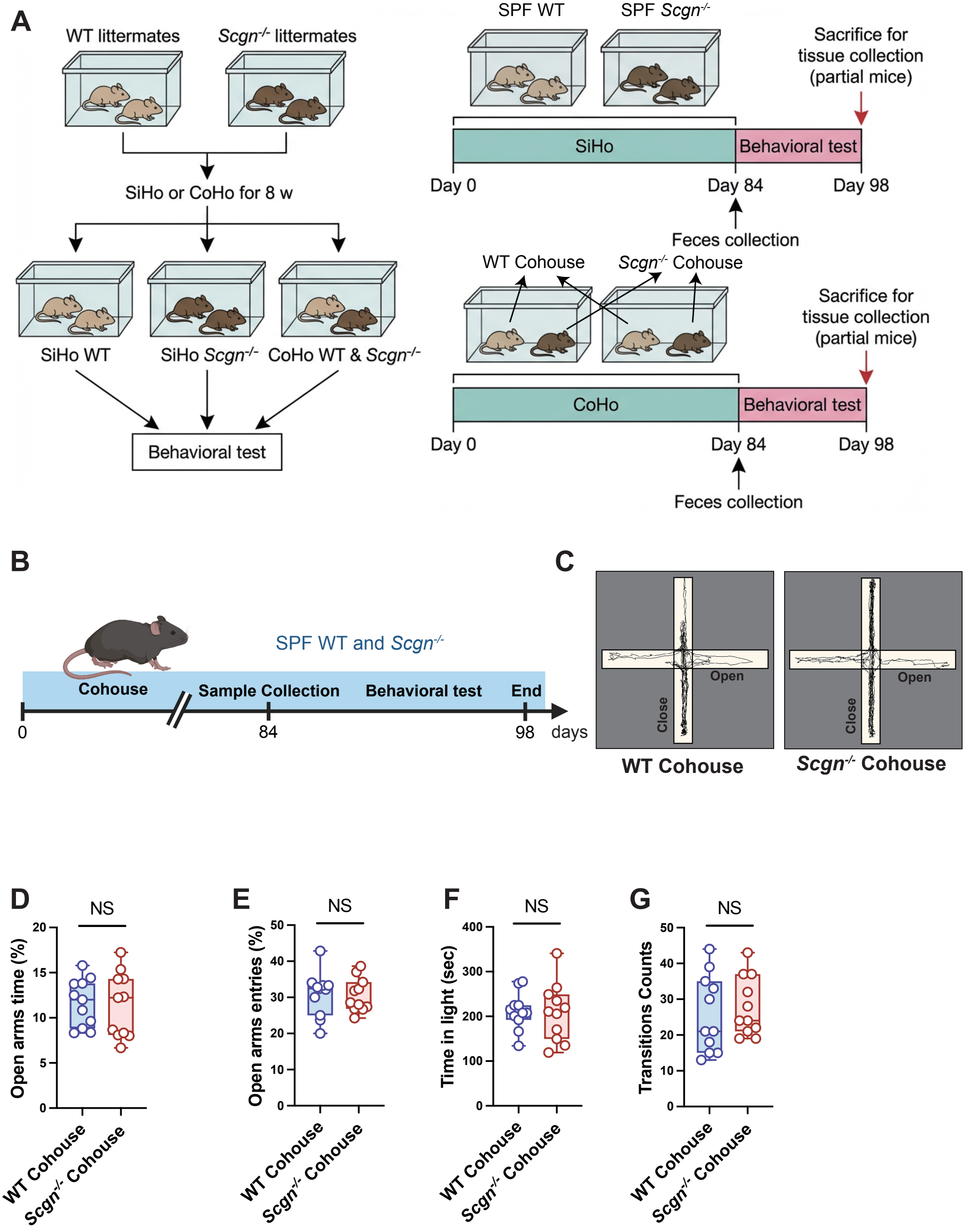

A
WT littermates
Scgn-/- littermates
SiHo or CoHo for 8 w
SiHo WT
SiHo Scgn-/-
CoHo WT & Scgn-/-
Behavioral test
SPF WT
SPF Scgn-/-
Sacrifice for tissue collection (partial mice)
SiHo
Behavioral test
Day 0
Day 84
Day 98
Feces collection
WT Cohouse
Scgn-/- Cohouse
CoHo
B
SPF WT and Scgn-/-
Cohouse
Sample Collection
Behavioral test
End
0
84
98
days
C
Open
Close
WT Cohouse
Scgn-/- Cohouse
D
NS
Open arms time (%)
E
Open arms entries (%)
F
Time in light (sec)
G
Transitions Counts
