# Supplementary material for: Butyrate Deficiency due to Microbial Dysbiosis Promotes Anxiety
Source: Research (Wash D C). 2026 Nov 9;9:1470. doi: 10.34133/research.1470 (PMC13745766; doi:10.34133/research.1470)
Supplement: Supplementary file 1 [file research.1470.f1.zip › sf5.pdf]

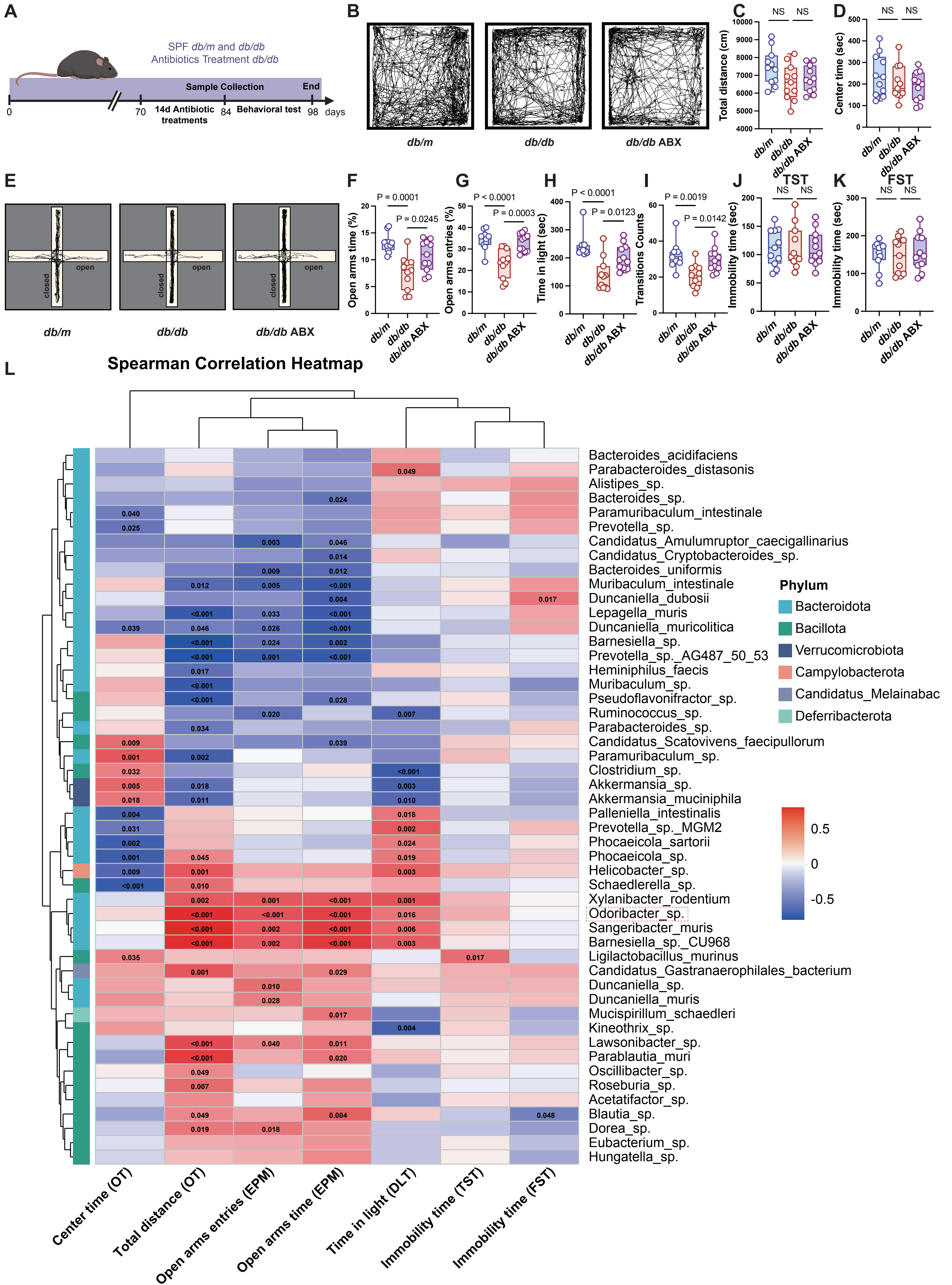

A
SPF db/m and db/db
Antibiotics Treatment db/db
Sample Collection
End
0
70
14d Antibiotic treatments
84
Behavioral test
98
days
B
db/m
db/db
db/db ABX
C
NS
NS
Total distance (cm)
D
NS
NS
Center time (sec)
E
closed
open
db/m
db/db
db/db ABX
F
P = 0.0001
P = 0.0245
Open arms time (%)
G
P < 0.0001
P = 0.0003
Open arms entries (%)
H
P < 0.0001
P = 0.0123
Time in light (sec)
I
P = 0.0019
P = 0.0142
Transitions Counts
J
TST
NS
NS
Immobility time (sec)
K
FST
NS
NS
Immobility time (sec)
L
Spearman Correlation Heatmap
Bacteroides_acidifaciens
Parabacteroides_distasonis
Alistipes_sp.
Bacteroides_sp.
Paramuribaculum_intestinale
Prevotella_sp.
Candidatus_Amulumruptor_caecigallinarius
Candidatus_Cryptobacteroides_sp.
Bacteroides_uniformis
Muribaculum_intestinale
Duncaniella_dubosii
Lepagella_muris
Duncaniella_muricolitica
Barnesiella_sp.
Prevotella_sp._AG487_50_53
Heminiphilus_faecis
Muribaculum_sp.
Pseudoflavonifractor_sp.
Ruminococcus_sp.
Parabacteroides_sp.
Candidatus_Scatovivens_faecipullorum
Paramuribaculum_sp.
Clostridium_sp.
Akkermansia_sp.
Akkermansia_muciniphila
Palleniella_intestinalis
Prevotella_sp._MGM2
Phocaeicola_sartorii
Phocaeicola_sp.
Helicobacter_sp.
Schaedlerella_sp.
Xylanibacter_rodentium
Odoribacter_sp.
Sangeribacter_muris
Barnesiella_sp._CU968
Ligilactobacillus_murinus
Candidatus_Gastranaerophilales_bacterium
Duncaniella_sp.
Duncaniella_muris
Mucispirillum_schaedleri
Kineothrix_sp.
Lawsonibacter_sp.
Parablautia_muri
Oscillibacter_sp.
Roseburia_sp.
Acetatifactor_sp.
Blautia_sp.
Dorea_sp.
Eubacterium_sp.
Hungatella_sp.
Phylum
Bacteroidota
Bacillota
Verrucomicrobiota
Campylobacterota
Candidatus_Melainabac
Deferribacterota
0.5
0
-0.5
Center time (OT)
Total distance (OT)
Open arms entries (EPM)
Open arms time (EPM)
Time in light (DLT)
Immobility time (TST)
Immobility time (FST)
