# Supplementary material for: Butyrate Deficiency due to Microbial Dysbiosis Promotes Anxiety
Source: Research (Wash D C). 2026 Nov 9;9:1470. doi: 10.34133/research.1470 (PMC13745766; doi:10.34133/research.1470)
Supplement: Supplementary file 1 [file research.1470.f1.zip › sf6.pdf]

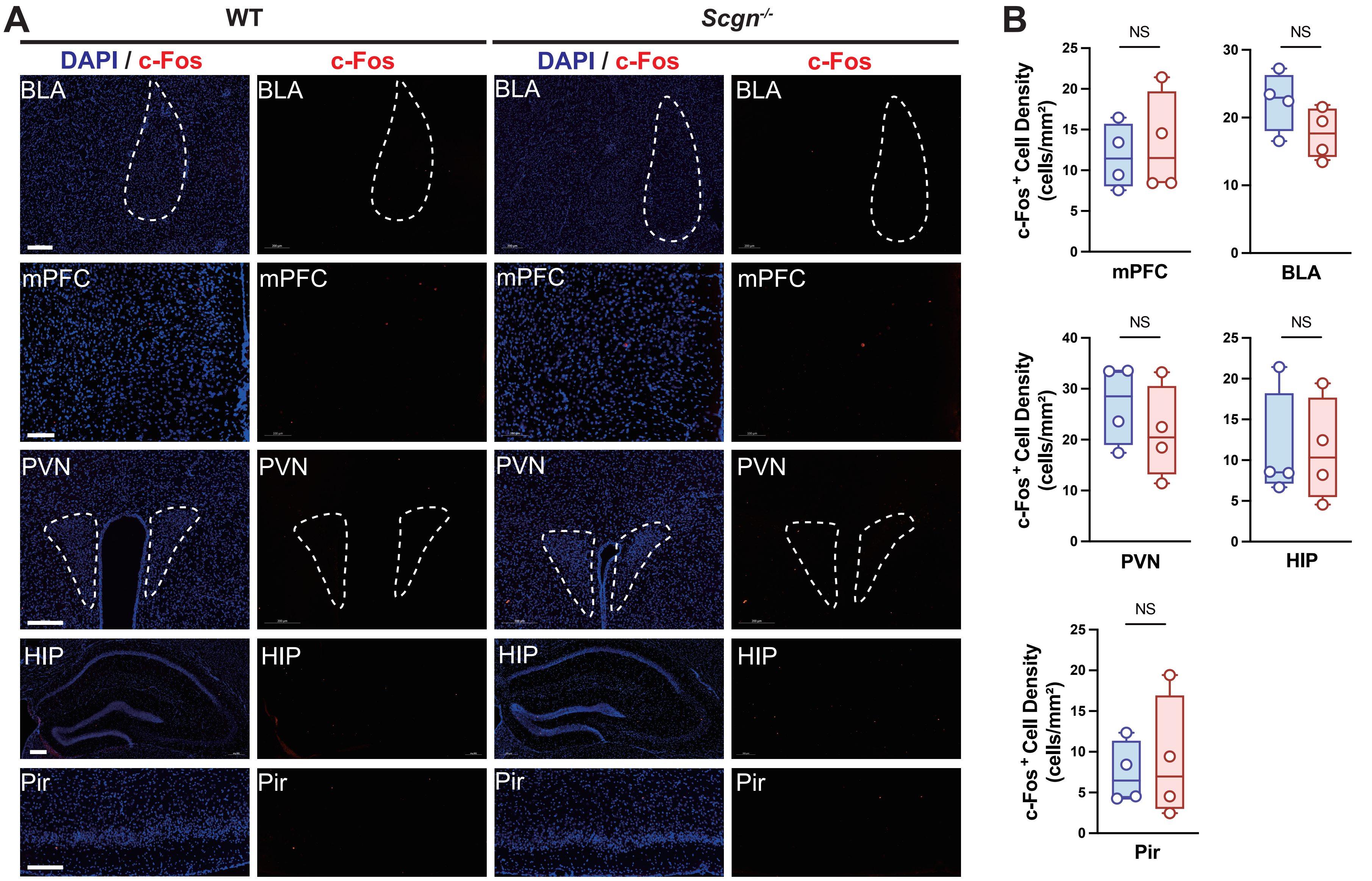

A
WT
Scgn-/-
DAPI / c-Fos
c-Fos
BLA
mPFC
PVN
HIP
Pir
B
NS
c-Fos + Cell Density (cells/mm²)
0
5
10
15
20
25
30
40
mPFC
BLA
PVN
HIP
Pir
