# Supplementary material for: Butyrate Deficiency due to Microbial Dysbiosis Promotes Anxiety
Source: Research (Wash D C). 2026 Nov 9;9:1470. doi: 10.34133/research.1470 (PMC13745766; doi:10.34133/research.1470)
Supplement: Supplementary file 1 [file research.1470.f1.zip › sf7.pdf]

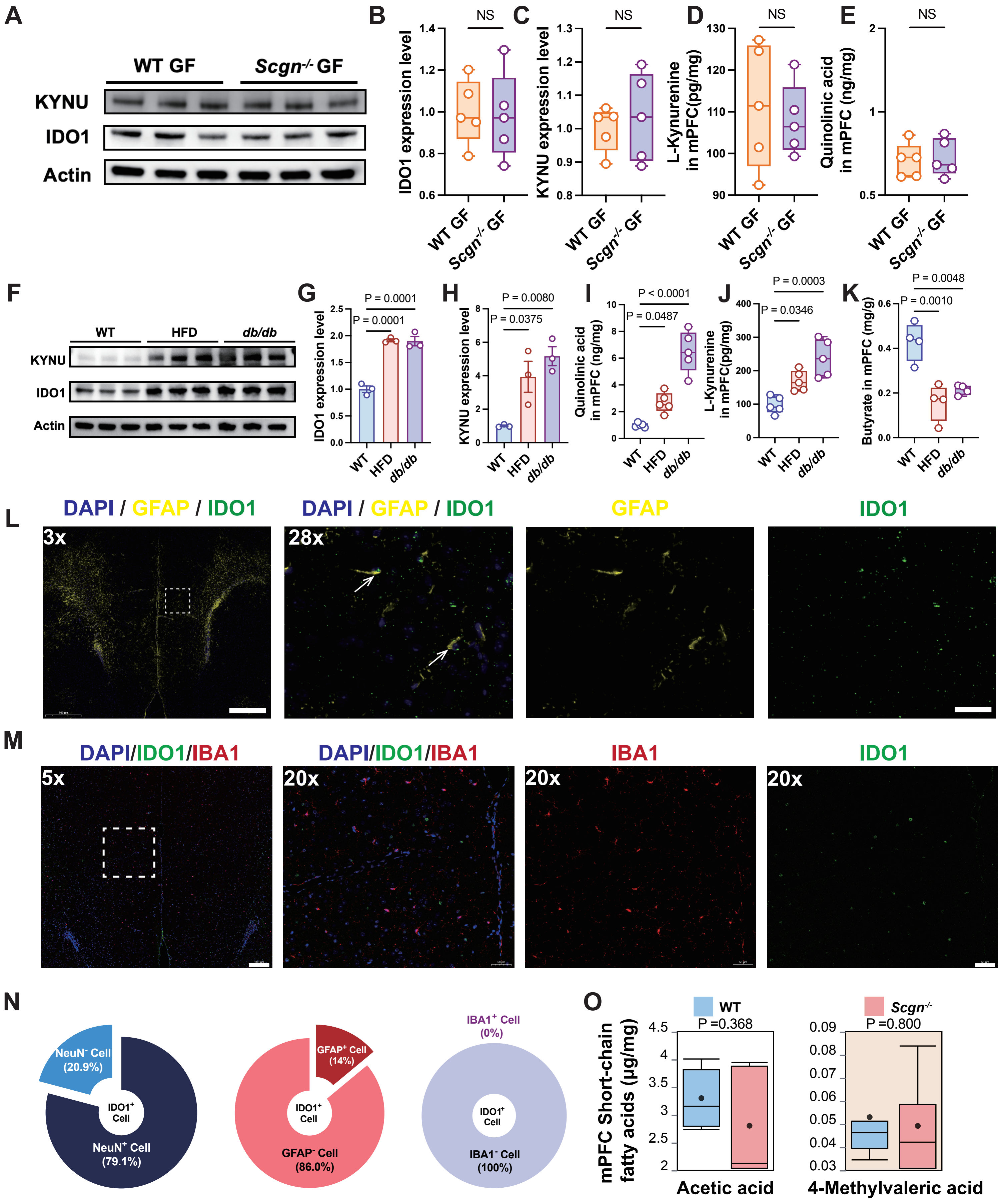
A
WT GF
Scgn-/- GF
KYNU
IDO1
Actin
B
NS
IDO1 expression level
WT GF
Scgn-/- GF
C
NS
KYNU expression level
D
NS
L-Kynurenine in mPFC(pg/mg)
E
NS
Quinolinic acid in mPFC (ng/mg)
F
WT
HFD
db/db
KYNU
IDO1
Actin
G
P = 0.0001
P = 0.0001
IDO1 expression level
H
P = 0.0080
P = 0.0375
KYNU expression level
I
P < 0.0001
P = 0.0487
Quinolinic acid in mPFC (ng/mg)
J
P = 0.0003
P = 0.0346
L-Kynurenine in mPFC(pg/mg)
K
P = 0.0048
P = 0.0010
Butyrate in mPFC (mg/g)
L
DAPI / GFAP / IDO1
3x
28x
GFAP
IDO1
M
DAPI/IDO1/IBA1
5x
20x
IBA1
IDO1
N
NeuN- Cell (20.9%)
IDO1+ Cell
NeuN+ Cell (79.1%)
GFAP+ Cell (14%)
GFAP- Cell (86.0%)
IBA1+ Cell (0%)
IBA1- Cell (100%)
O
WT
Scgn-/-
mPFC Short-chain fatty acids (μg/mg)
P =0.368
P =0.800
Acetic acid
4-Methylvaleric acid
