# Supplementary material for: Butyrate Deficiency due to Microbial Dysbiosis Promotes Anxiety
Source: Research (Wash D C). 2026 Nov 9;9:1470. doi: 10.34133/research.1470 (PMC13745766; doi:10.34133/research.1470)
Supplement: Supplementary file 1 [file research.1470.f1.zip › sf8.pdf]

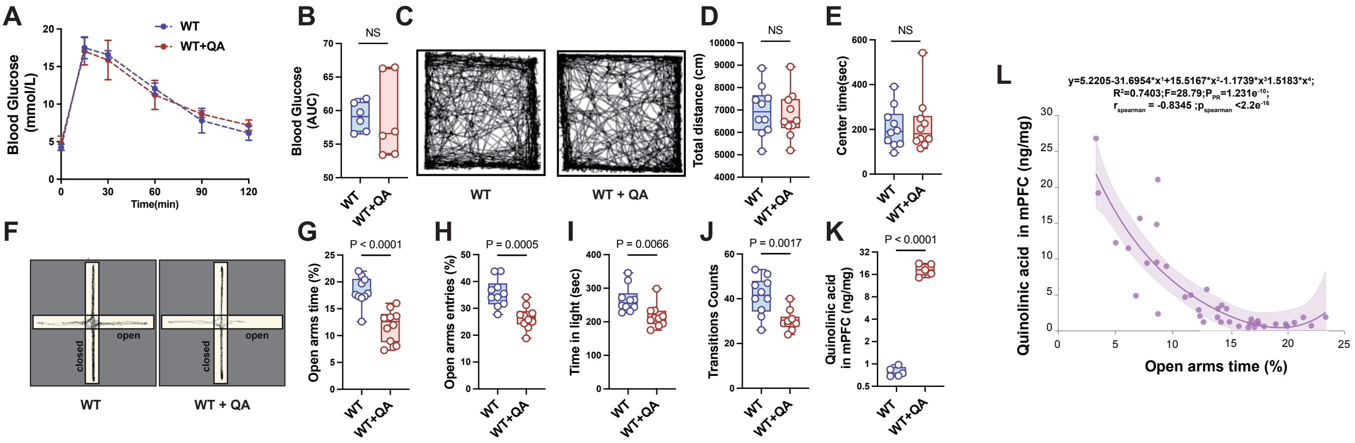

A
B
C
D
E
F
G
H
I
J
K
L
WT
WT+QA
Blood Glucose (mmol/L)
Time(min)
Blood Glucose (AUC)
NS
WT + QA
Total distance (cm)
Center time(sec)
open
closed
Open arms time (%)
P < 0.0001
Open arms entries (%)
P = 0.0005
Time in light (sec)
P = 0.0066
Transitions Counts
P = 0.0017
Quinolinic acid in mPFC (ng/mg)
y=5.2205-31.6954*x¹+15.5167*x²-1.1739*x³1.5183*x⁴;
R²=0.7403;F=28.79;P_PR=1.231e-10;
r_spearman = -0.8345 ;p_spearman <2.2e-16
Quinolinic acid in mPFC (ng/mg)
Open arms time (%)
