# Supplementary material for: Butyrate Deficiency due to Microbial Dysbiosis Promotes Anxiety
Source: Research (Wash D C). 2026 Nov 9;9:1470. doi: 10.34133/research.1470 (PMC13745766; doi:10.34133/research.1470)
Supplement: Supplementary file 1 [file research.1470.f1.zip › sf9.pdf]

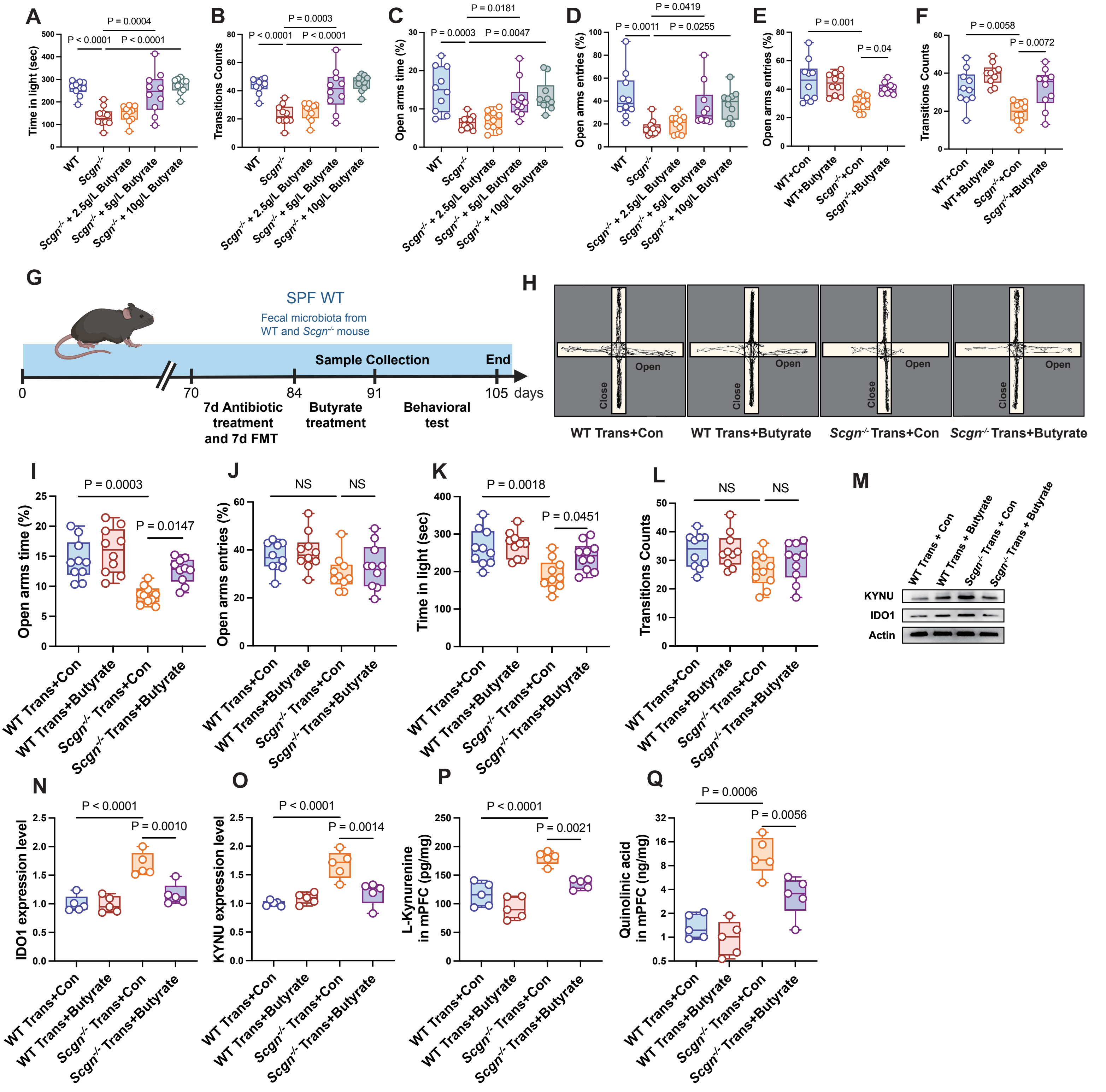
A
Time in light (sec)
P < 0.0001
P = 0.0004
P < 0.0001
WT
Scgn-/-
Scgn-/- + 2.5g/L Butyrate
Scgn-/- + 5g/L Butyrate
Scgn-/- + 10g/L Butyrate
B
Transitions Counts
P < 0.0001
P = 0.0003
P < 0.0001
C
Open arms time (%)
P = 0.0003
P = 0.0181
P = 0.0047
D
Open arms entries (%)
P = 0.0011
P = 0.0419
P = 0.0255
E
Open arms entries (%)
P = 0.001
P = 0.04
WT+Con
WT+Butyrate
Scgn-/-+Con
Scgn-/-+Butyrate
F
Transitions Counts
P = 0.0058
P = 0.0072
G
SPF WT
Fecal microbiota from
WT and Scgn-/- mouse
Sample Collection
End
0
70
84
91
105 days
7d Antibiotic treatment and 7d FMT
Butyrate treatment
Behavioral test
H
Open
Close
WT Trans+Con
WT Trans+Butyrate
Scgn-/- Trans+Con
Scgn-/- Trans+Butyrate
I
Open arms time (%)
P = 0.0003
P = 0.0147
J
Open arms entries (%)
NS
NS
K
Time in light (sec)
P = 0.0018
P = 0.0451
L
Transitions Counts
NS
NS
M
WT Trans + Con
WT Trans + Butyrate
Scgn-/- Trans + Con
Scgn-/- Trans + Butyrate
KYNU
IDO1
Actin
N
IDO1 expression level
P < 0.0001
P = 0.0010
O
KYNU expression level
P < 0.0001
P = 0.0014
P
L-Kynurenine in mPFC (pg/mg)
P < 0.0001
P = 0.0021
Q
Quinolinic acid in mPFC (ng/mg)
P = 0.0006
P = 0.0056
