# Supplementary material for: Butyrate Deficiency due to Microbial Dysbiosis Promotes Anxiety
Source: Research (Wash D C). 2026 Nov 9;9:1470. doi: 10.34133/research.1470 (PMC13745766; doi:10.34133/research.1470)
Supplement: Supplementary file 1 [file research.1470.f1.zip › sf10.pdf]

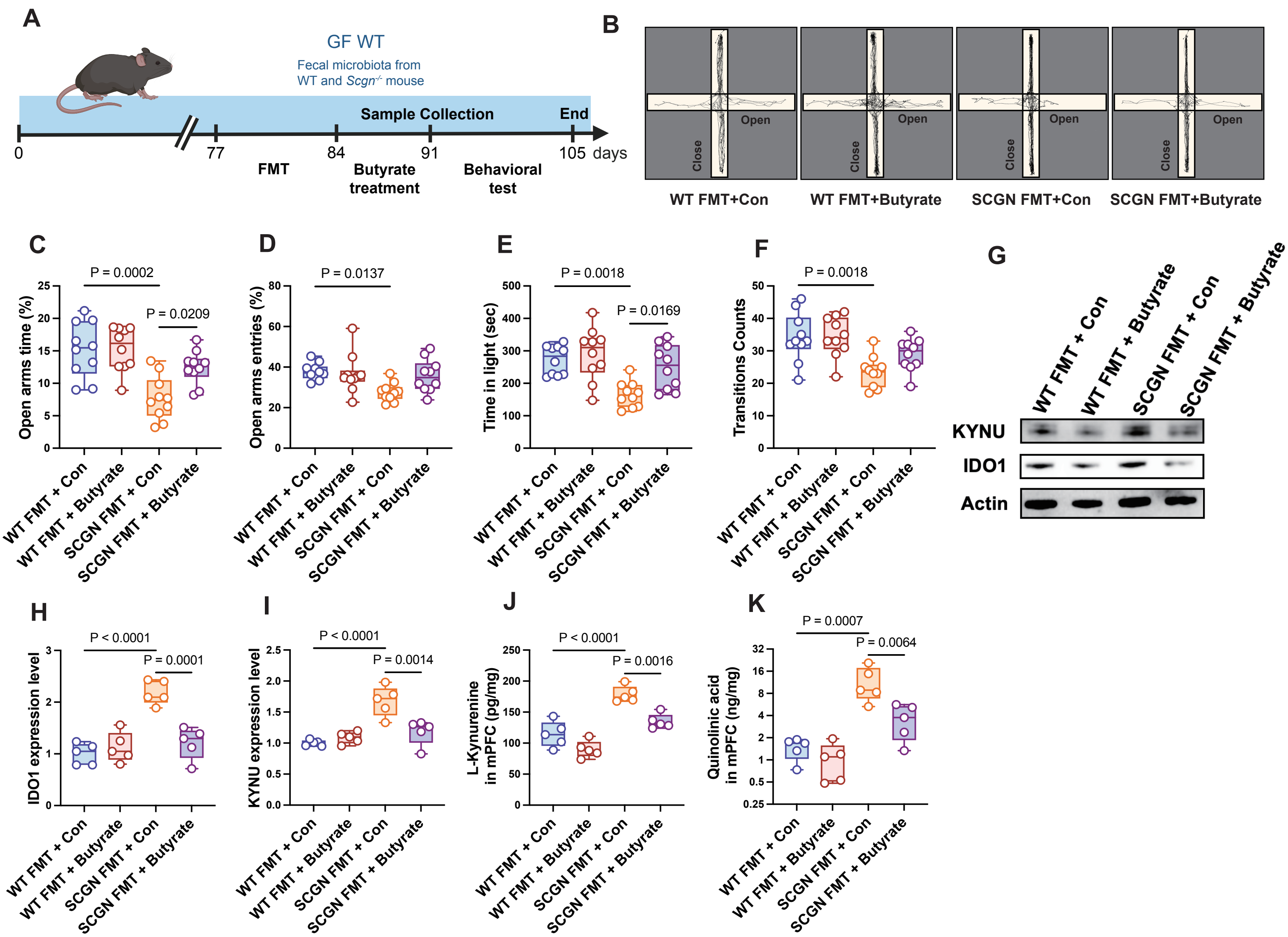
A
GF WT
Fecal microbiota from
WT and $Scgn^{-/-}$ mouse
Sample Collection
End
0
77
84
91
105 days
FMT
Butyrate treatment
Behavioral test
B
Open
Close
WT FMT+Con
WT FMT+Butyrate
SCGN FMT+Con
SCGN FMT+Butyrate
C
Open arms time (%)
P = 0.0002
P = 0.0209
D
Open arms entries (%)
P = 0.0137
E
Time in light (sec)
P = 0.0018
P = 0.0169
F
Transitions Counts
P = 0.0018
G
KYNU
IDO1
Actin
WT FMT + Con
WT FMT + Butyrate
SCGN FMT + Con
SCGN FMT + Butyrate
H
IDO1 expression level
P < 0.0001
P = 0.0001
I
KYNU expression level
P < 0.0001
P = 0.0014
J
L-Kynurenine in mPFC (pg/mg)
P < 0.0001
P = 0.0016
K
Quinolinic acid in mPFC (ng/mg)
P = 0.0007
P = 0.0064
