# Supplementary material for: Butyrate Deficiency due to Microbial Dysbiosis Promotes Anxiety
Source: Research (Wash D C). 2026 Nov 9;9:1470. doi: 10.34133/research.1470 (PMC13745766; doi:10.34133/research.1470)
Supplement: Supplementary file 1 [file research.1470.f1.zip › sf11.pdf]

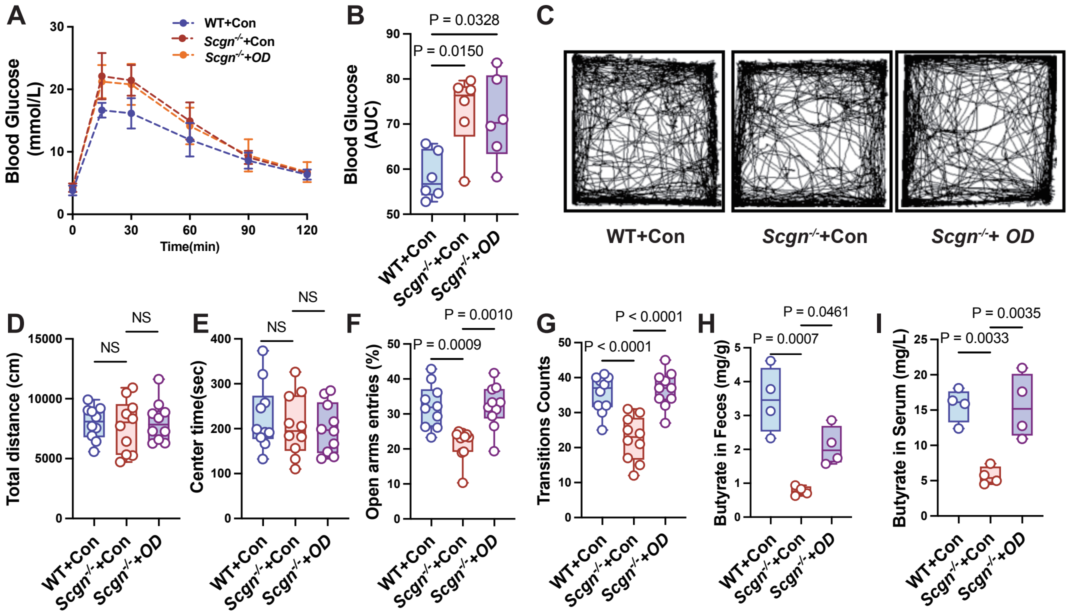

A
B
C
D
E
F
G
H
I
WT+Con
Scgn-/-+Con
Scgn-/-+OD
Blood Glucose (mmol/L)
Time(min)
Blood Glucose (AUC)
P = 0.0328
P = 0.0150
WT+Con
Scgn-/-+Con
Scgn-/-+ OD
Total distance (cm)
NS
NS
Center time(sec)
NS
NS
Open arms entries (%)
P = 0.0010
P = 0.0009
Transitions Counts
P < 0.0001
P < 0.0001
Butyrate in Feces (mg/g)
P = 0.0461
P = 0.0007
Butyrate in Serum (mg/L)
P = 0.0035
P = 0.0033
