# Supplementary material for: Butyrate Deficiency due to Microbial Dysbiosis Promotes Anxiety
Source: Research (Wash D C). 2026 Nov 9;9:1470. doi: 10.34133/research.1470 (PMC13745766; doi:10.34133/research.1470)
Supplement: Supplementary file 1 [file research.1470.f1.zip › sf12.pdf]

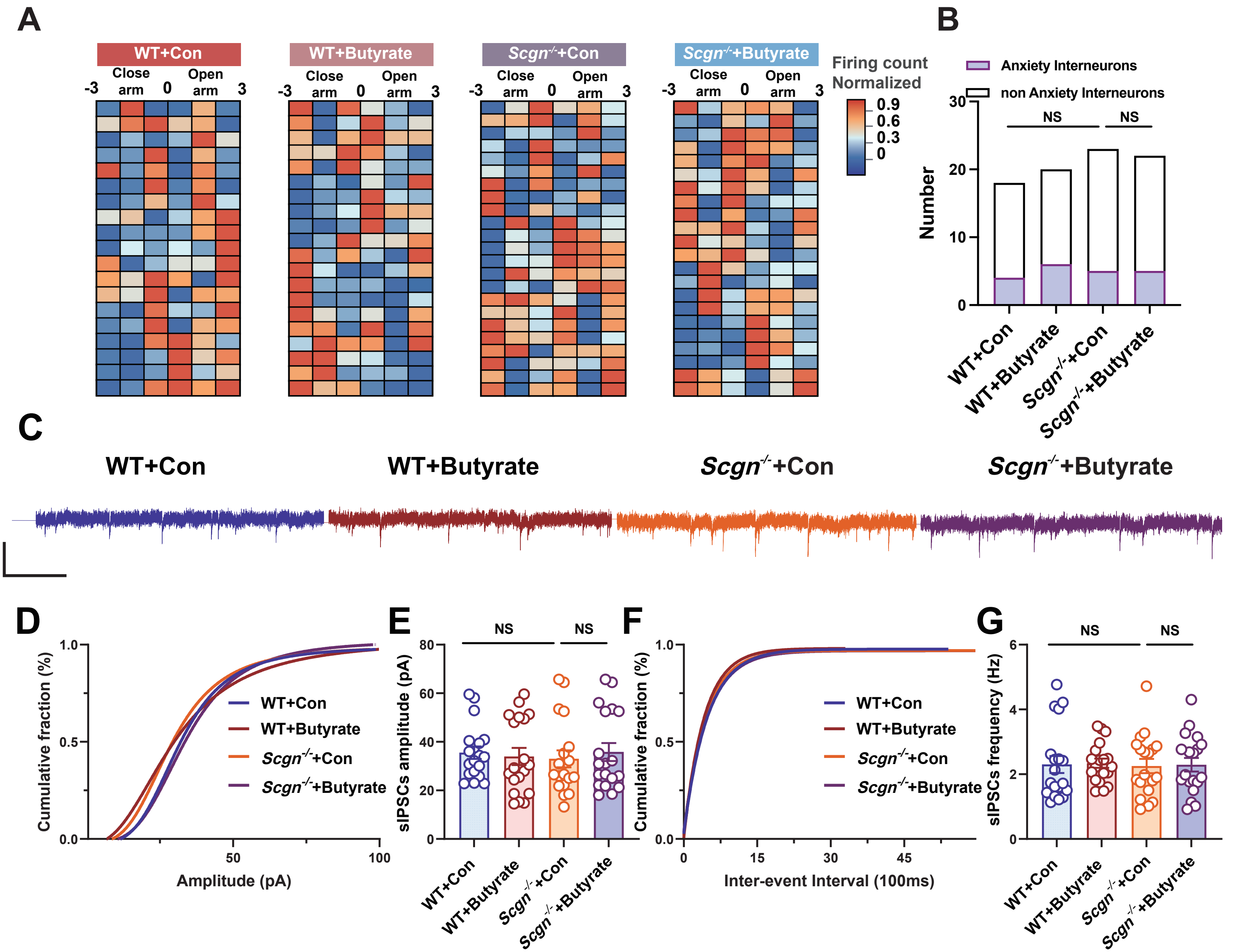
A
WT+Con
WT+Butyrate
Scgn-/-+Con
Scgn-/-+Butyrate
Close arm
Open arm
-3
0
3
Firing count Normalized
0.9
0.6
0.3
0
B
Anxiety Interneurons
non Anxiety Interneurons
NS
NS
Number
C
WT+Con
WT+Butyrate
Scgn-/-+Con
Scgn-/-+Butyrate
D
Cumulative fraction (%)
Amplitude (pA)
E
sIPSCs amplitude (pA)
NS
NS
F
Cumulative fraction (%)
Inter-event Interval (100ms)
G
sIPSCs frequency (Hz)
NS
NS
