# Supplementary material for: Butyrate Deficiency due to Microbial Dysbiosis Promotes Anxiety
Source: Research (Wash D C). 2026 Nov 9;9:1470. doi: 10.34133/research.1470 (PMC13745766; doi:10.34133/research.1470)
Supplement: Supplementary file 1 [file research.1470.f1.zip › Supplementary Materials.docx]

SUPPLEMENTARY MATERIALS

**Supplementary Materials**

Fig. S1 Metabolic phenotypes of diabetic SPF db/db mice and *Scgn^-/-^* mice.

(A) Body weight comparison between SPF *db/m* and *db/db* mice. ( n = 10 mice per group).

(B) Blood glucose levels in SPF *db/m* and *db/db* mice. (n = 10 mice per group).

(C) Blood glucose levels during glucose tolerance test in SPF WT and *Scgn^-/-^* mice. (n = 8 mice per group).

(D) Area under the curve (AUC) for blood glucose during glucose tolerance test in SPF WT and *Scgn^-/-^* mice. (n = 8 mice per group).

(E) Blood glucose levels in SPF WT， HFD, and *db/db* mice. ( n = 6 mice per group).

(F) Body weight comparison between SPF WT, HFD, and *db/db* mice. ( n = 6 mice per group).

(G) Representative movement trajectories of SPF WT, HFD, and *db/db* mice during the Open Field Test are depicted.

(H) The total distance traveled by SPF WT, HFD, and *db/db* mice in the Open Field Test is quantified( n = 6 mice per group).

(I) The duration spent in the center area during the Open Field Test by SPF WT, HFD, and *db/db* mice is reported( n = 6 mice per group).

(J) Representative movement trajectories for SPF WT, HFD, and *db/db* mice during the Elevated Plus Maze Test are presented.

(K) The duration spent in the open arms of the Elevated Plus Maze Test by SPF WT, HFD, and *db/db* mice is quantitatively assessed ( n = 6 mice per group).

(L) The number of entries into the Elevated Plus Maze Test by SPF WT, HFD, and db/db mice is documented ( n = 6 mice per group).

(M) The duration spent in the Light compartment of the Light-Dark Box Test by SPF WT, HFD, and *db/db* mice is evaluated( n = 6 mice per group).

**(**N**)** The counts of compartment transitions observed in SPF WT, HFD, and *db/db* mice during the Light-Dark Box Test are reported ( n = 6 mice per group).

Data are presented as box plots showing median and interquartile range; individual data points represent biological replicates. NS, not significant.

Statistical analysis:

Unpaired two-tailed Student’s t-test:A, B, D, E, F, and H-L.

Fig. S2 Behavioral Assessment in SPF WT and *Scgn^-/-^* Mice

(A) Escape latency during the Morris water maze training period in SPF WT and *Scgn^-/-^* mice. (n = 11 mice per group).

(B) Time spent in the target quadrant during probe testing in the Morris Water Maze Test of SPF WT and *Scgn^-/-^* mice. (n = 11 mice per group).

(C) Number of platform crossings during the probe test in the Morris Water Maze Test of SPF WT and *Scgn^-/-^* mice. (n = 11 mice per group).

(D) Discrimination index in the Novel Object Recognition (NOR) test of SPF WT and *Scgn^-/-^* mice. (n = 11 mice per group).

(E) Percentage of spontaneous alternation performance (SAP) in the Y-Maze Test of SPF WT and *Scgn^-/-^* mice. (n = 11 mice per group).

(F) Spontaneous alternation behavior (SAB) in the Y-Maze Test of SPF WT and *Scgn^-/-^* mice. (n = 11 mice per group).

(G) Nest building scores in SPF WT and *Scgn^-/-^* mice. (n = 11 mice per group).

(H) Volcano plot showing DEGs for SPF WT and Scgn-/- colon RNA-seq (bulk RNA-seq; DESeq2). Numbers indicate down-regulated (n = 241) and up-regulated (n = 137) genes(P value < 0.05; |fold change| ≥ 1 ); exemplar genes are labeled. Each point represents one gene.

(I) Gene Ontology (GO) enrichment analysis of differentially expressed genes in SPF WT and *Scgn^-/-^* mice colon sample, showing terms related to biological processes (BP), cellular components (CC), and molecular functions (MF). Bubble size represents gene count, and color indicates GO category.

(J) KEGG bubble plot of the eight significant pathways (BH-adjusted p < 0.05). Dot size indicates the number of DEGs in the pathway; color encodes adjusted p-value; the x-axis shows the gene ratio.

Data are presented as box plots showing median(A-F) and interquartile range and mean ± SEM(G); individual data points represent biological replicates. NS, not significant.

Statistical analysis:

Unpaired two-tailed Student’s t-test: B-G.

Fig. S3 Social and Repetitive Behavioral Assessment in SPF and GF WT and *Scgn^-/-^* Mice with Butyrate Treatment

(A, E) Sniffing time for social novelty (S1 vs S2) in the Three-Chamber Social Interaction Test of SPF WT , *Scgn^-/-^* , and butyrate-treated *Scgn^-/-^* mice (A), and GF WT , *Scgn^-/-^* and butyrate-treated *Scgn^-/-^* mice (E). (n = 10 mice per group).

(B, F) Preference index (S1-S2) based on sniffing time in the Three-Chamber Social Interaction Test of SPF WT , *Scgn^-/-^* and butyrate-treated *Scgn^-/-^* mice (B), and GF WT , *Scgn^-/-^* and butyrate-treated *Scgn^-/-^* mice (F). (n = 10 mice per group).

(C, G) Duration of self-grooming in SPF WT , *Scgn^-/-^* and butyrate-treated *Scgn^-/-^* mice (C), and GF WT , *Scgn^-/-^* and butyrate treated *Scgn^-/-^* mice (G). ( n = 10 mice per group).

(D, H) Number of marbles buried in Marble Burying Test for SPF WT , *Scgn^-/-^* and butyrate-treated *Scgn^-/-^* mice (D), and GF WT , *Scgn^-/-^* and butyrate-treated *Scgn^-/-^* mice (H). (n = 10 mice per group).

Data are presented as box plots showing median and interquartile range; individual data points represent biological replicates. NS, not significant.

Statistical analysis:

One-way analysis of variance (ANOVA) followed by Tukey’s post hoc test: B-D and F-H.

Paired two-tailed Student’s t-test: A and E.

Fig. S4 Behavioral and Gut Microbiota Assessment in WT and *Scgn^-/-^* Mice after Co-housing

(A)Experimental timeline diagram of co-housing WT and *Scgn^-/-^* mice.

Upon weaning, WT and *Scgn^-/-^* mice were co-housed in the same cages. Fecal and brain samples were collected at 84 days of age to assess gut microbiota composition. Within the following two weeks (days 84 to 98), a comprehensive battery of behavioral tests was performed in the following order: Elevated Plus Maze Test and Light-Dark Box Test.

(B) Experimental timeline diagram of co-housed WT and *Scgn*^-/-^ mice.

Upon weaning, WT and *Scgn*^-/-^ mice were co-housed in the same cages. At 84 days of age, Fecal and brain samples were collected to assess gut microbiota composition. Within the following two weeks (days 84 to 98), a comprehensive battery of behavioral tests was performed in the following order: Elevated Plus Maze Test and Light-Dark Box Test.

(C) Representative movement trajectories of SPF WT and *Scgn*^-/-^ mice after co-housing in the Elevated Plus Maze Test.

(D, E) Open-arm duration (D) and open-arm entries (E) of SPF WT and *Scgn*^-/-^ mice after co-housing in the Elevated Plus Maze Test. (n = 11 mice per group).

(F, G) Light compartment duration (F) and Compartment transition counts (G) of SPF WT and *Scgn*^-/-^ mice after co-housing in the Elevated Plus Maze Test. (n = 11 mice per group).

Data are presented as box plots showing median and interquartile range; individual data points represent biological replicates. NS, not significant.

Statistical analysis:

Unpaired two-tailed Student’s t-test: C-F.

Fig. S5 Behavioral test of SPF *db/m*, *db/db*, and antibiotic-treated *db/db* mice.

(A) Experimental timeline diagram of SPF *db/m*, *db/db*, and antibiotic-treated *db/db* mice. Upon weaning, mice were individually housed in single cages. Antibiotic-treated cohorts received drinking water supplemented with Neomycin (1 g/L), Metronidazole (1 g/L), Ampicillin (1 g/L), and Vancomycin (500 mg/L) from day 70 to 84. At 84 days of age, medial prefrontal cortex (mPFC) and plasma samples were collected. Behavioral tests were then conducted over the following two weeks in the following order: Open Field Test, Elevated Plus Maze Test, Light-Dark Box Test, Tail Suspension Test, and Forced Swim Test.

(B) Representative movement traces in the Open field test for SPF *db/m*, *db/db*, and antibiotic-treated *db/db* mice.

(C-D) Total distance traveled by SPF *db/m*, *db/db*, and antibiotic-treated *db/db* mice in the Open Field Test. (n = 11 mice per group).

(E) Representative movement trajectories of SPF *db/m*, *db/db*, and antibiotic-treated *db/db* mice in the Elevated Plus Maze Test.

(F-G) Open-arm duration and open-arm entries of SPF *db/m*, *db/db*, and antibiotic- treated *db/db* mice in the Elevated Plus Maze Test. (n = 11 mice per group).

(H-I) Light compartment duration and Compartment transition counts of SPF *db/m*, *db/db*, and antibiotic-treated *db/db* mice during the Light-Dark Box Test. (n = 11 mice per group).

(J-K) Immobility time of SPF *db/m*, *db/db*, and antibiotic-treated *db/db* mice during the Tail Suspension Test(J) and the Forced Swim Test(K). (n = 11 mice per group).

(L) Spearman correlation heatmap of microbial taxa with behavioral parameters (total distance, open-arm entries, open-arm time, center time, time in light, immobility time in EPM, DLT, and FST tests). Each row represents a different bacterial species or genus, and the colors indicate correlation strength (blue:negative correlation, red:positive correlation).

Data are presented as box plots showing median and interquartile range; individual data points represent biological replicates. NS, not significant.

Statistical analysis:One-way analysis of variance (ANOVA) followed by Tukey’s post hoc test : C-D and F-K.

Spearman correlation analysis: L.

Fig. S6 Basal c-Fos expression shows no overt activation in SPF WT and *Scgn^-/-^* mice at rest

(A) Representative c-Fos immunofluorescence images from the medial prefrontal cortex (mPFC), basolateral amygdala (BLA), paraventricular nucleus (PVN), hippocampus (HIP), and piriform cortex (Pir) of SPF WT and *Scgn^-/-^* mice under resting (basal) conditions (red: c-Fos; blue: DAPI). Scale bars are 100 μm for mPFC and 200 μm for the other regions.

(B) A comparison of the number of c-Fos^+^ cells across different brain regions in SPF WT and *Scgn^-/-^* mice is provided (n = 4 mice per group).

Data are presented as box plots showing the median and interquartile range; individual data points represent biological replicates. NS, not significant.

Statistical analysis:

Unpaired two-tailed Student’s t-test: B.

Fig. S7 Localization of IDO1 and levels of SCFAs in the mPFC and tryptophan metabolism in the mPFC of GF and *Scgn^-/-^* mice

(A) Representative western blots illustrating the enzymes related to tryptophan metabolism in the mPFC of GF WT and *Scgn^-/-^* mice.

(B-C) The protein expression levels of IDO1 (B) and KYNU (C) were assessed in the mPFC of GF WT and *Scgn^-/-^* mice (n = 5 mice per group).

(D-E) Concentrations of L-kynurenine (D) and quinolinic acid (QA) (E) in the mPFC of GF WT and *Scgn^-/-^* mice (n = 5 mice per group).

(F) Representative western blots depict the expression of enzymes associated with tryptophan metabolism in the mPFC of SPF WT, HFD, and *db/db* mice.

(G-H) Protein expression levels of IDO1 (G) andKYNU (H) in SPF WT, HFD and db/db mice (n = 3 mice per group).

(I-J) Quinolinic acid (QA) (I) and L-kynurenine (J) concentrations in the mPFC of SPF WT, HFD, and *db/db* mice (n = 5 mice per group).

(K) Butyrate concentration in the mPFC of SPF WT, HFD, and *db/db* mice(n = 4 mice per group).

(L) Representative images of the mouse medial prefrontal cortex (mPFC) showing DAPI (blue), IDO1 (green, in situ hybridization), and GFAP (yellow, immunostaining by antibody). Images are shown at 3 × ( scale bar = 500 μm) and 28 × ( scale bar = 50 μm) magnifications, with single channels for GFAP and IDO1.

(M) Representative images of the mouse medial prefrontal cortex (mPFC) showing DAPI (blue), IDO1 (green, in situ hybridization), and IBA1 (red, immunostaining by antibody). Images are shown at 5 × ( scale bar = 200 μm) and 20 × ( scale bar = 50 μm) magnifications, with single channels for IBA1 and IDO1.

(N) Quantification of IDO1 expression in different cell types. Pie charts represent the percentage of IDO1^+^ cells that co-localize with GFAP^+^ astrocytes, NEUN^+^ neurons, and IBA1+ microglia. The data show that the majority of IDO1+ cells are IBA1^+^ microglia, with a smaller percentage in GFAP^+^ astrocytes and NEUN^+^ neurons.

(O) Levels of acetic acid and 4-methylvaleric acid in the mPFC of SPF WT and *Scgn^-/-^* mice. (n = 4 mice per group).

Data are presented as box plots showing median and interquartile range; individual data points represent biological replicates. NS, not significant.

Statistical analysis:

Unpaired two-tailed Student’s t-test: B-E and O

One-way analysis of variance (ANOVA) followed by Tukey’s post hoc test : G-K.

Fig. S8 Quinolinic acid induced anxiety-like behavior.

(A) Blood glucose levels during the glucose tolerance test in SPF WT and WT+QA mice. (n = 6 mice per group).

(B) Area under the curve (AUC) for blood glucose during the glucose tolerance test in SPF WT and WT+QA mice. (n = 6 mice per group).

(C) Representative movement trajectories of SPF WT and WT+QA mice during the Open Field Test are depicted.

(D) The total distance traveled by SPF WT and WT+QA mice in the Open Field Test is quantified ( n = 10 mice per group).

(E) The duration spent in the center area during the Open Field Test by SPF WT and WT+QA mice is reported ( n = 10 mice per group).

(F) Representative movement trajectories for SPF WT and WT+QA mice during the Elevated Plus Maze Test are presented.

(G) The duration spent in the open arms of the Elevated Plus Maze Test by SPF WT and WT+QA mice is quantitatively assessed ( n = 10 mice per group).

(H) The number of entries into the Elevated Plus Maze Test by SPF WT and WT+QA mice is documented ( n =10 mice per group).

(I) The duration spent in the Light compartment of the Light-Dark Box Test by SPF WT and WT+QA mice is evaluated( n = 10 mice per group).

(J) Compartment transition counts during the Light-Dark Box Test in SPF WT and WT+QA mice ( n = 10 mice per group).

(K) Quinolinic acid (QA) concentrations in the mPFC of SPF WT and WT+QA mice (n = 5 mice per group).

(L) Correlation analysis between QA levels in the mPFC and the percentage of time spent in the open arms of the maze during Elevated Plus Maze Test.

Data are presented as box plots showing median and interquartile range; individual data points represent biological replicates. NS, not significant. Statistical analysis:

Unpaired two-tailed Student’s t-test: A-B and D-E and G-K

Spearman correlation with linear regression fit: L.

Fig. S9 Butyrate reverses anxiety-like behavior and kynurenine pathway changes induced by SCGN-deficient microbiota

(A, B) Light compartment duration(A) and compartment transition counts (B) of gradient butyrate-treated and untreated SPF WT and *Scgn*^-/-^ and *Scgn*^-/-^ +2.5-10g/L Butyrate mice in the Light-Dark Box Test. (n = 10 mice per group)

(C, D) Open arm duration (C) and open arm entries (D) of gradient butyrate-treated and untreated SPF WT and *Scgn*^-/-^ and *Scgn*^-/-^ +2.5-10g/L Butyrate mice in the Elevated Plus Maze Test. (n = 10mice per group)

(E, F) Open arm duration (E) and open arm entries (F) of butyrate treated and untreated SPF WT and from SPF WT and *Scgn*^-/-^ mice in the Elevated Plus Maze Test. (n = 10 mice per group)

(G) Experimental timeline diagram of butyrate administration in SPF WT and *Scgn*^-/-^ mice. Upon weaning, mice were individually housed in single cages. At 70 days of age, SPF WT mice received fecal microbiota from WT or *Scgn^-/-^* mice after 7 days of antibiotic treatment. At 84 days of age, experimental mice received one week of butyrate treatment by replacing their drinking water with water containing 5 g/L butyrate. At 91 days of age, medial prefrontal cortex (mPFC) samples were collected. Behavioral tests were then conducted over the following two weeks in the following order: Elevated Plus Maze Test and Light-Dark Box Test.

(H) Representative movement trajectories of butyrate-treated and untreated SPF WT mice following antibiotic treatment and FMT from SPF WT and *Scgn*^-/-^ donors and *Scgn*^-/-^ mice in the Elevated Plus Maze Test. (n = 10mice per group).

(I-J) Open arm duration (I) and open arm entries (J) of butyrate-treated and untreated SPF WT following antibiotic treatment and FMT from SPF WT and *Scgn*^-/-^ donors and *Scgn*^-/-^ mice in the Elevated Plus Maze Test. (n = 10 mice per group)

(K-L) Light compartment duration (K) and compartment transition counts (L) of butyrate-treated and untreated SPF WT following antibiotic treatment and FMT from SPF WT and *Scgn*^-/-^ donors and *Scgn*^-/-^ mice in the Light-Dark Box Test. (n = 10 mice per group)

(M) Representative western blots of tryptophan metabolism-related enzymes in the mPFC of butyrate-treated and untreated SPF WT following antibiotic treatment and FMT from SPF WT and *Scgn*^-/-^ donors and *Scgn*^-/-^ mice.

(N-O) Quantification of IDO1 (N) and KYNU (O)protein expression levels in the mPFC. (n = 5 mice per group)

(P-Q) Concentration of L-kynurenine (P) and quinolinic acid (Q) in the mPFC of butyrate-treated and untreated SPF WT and *Scgn*^-/-^ mice (n = 5 mice per group).

Data are presented as box plots showing median and interquartile range; individual data points represent biological replicates. NS, not significant.

Statistical analysis:

One-way analysis of variance (ANOVA) followed by Tukey’s post hoc test: A-F, I-L, and N-Q.

Fig. S10 Butyrate reverses anxiety-like behavior and kynurenine pathway changes induced by SCGN-deficient microbiota

(A) Experimental timeline diagram of butyrate administration in SPF WT and *Scgn*^-/-^ mice. Upon weaning, mice were individually housed in single cages. At 77 days of age, GF WT mice received fecal microbiota from WT or *Scgn^-/-^* mice. At 84 days of age, experimental mice received one week of butyrate treatment by replacing their drinking water with water containing 5 g/L butyrate. At 91 days of age, medial prefrontal cortex (mPFC) samples were collected. Behavioral tests were then conducted over the next two weeks in this order: Elevated Plus Maze Test and Light-Dark Box Test.

(B) Representative movement trajectories of butyrate-treated and untreated GF WT following FMT from SPF WT and *Scgn*^-/-^ donors and *Scgn*^-/-^ mice in the Elevated Plus Maze Test. (n = 10 mice per group).

(C–D) Open arm duration (C) and open arm entries (D) of butyrate-treated and untreated GF WT following FMT from SPF WT and *Scgn*^-/-^ donors and *Scgn*^-/-^ mice in the Elevated Plus Maze Test. (n = 10 mice per group)

(E-F) Light compartment duration (E) and compartment transition counts (F) of butyrate-treated and untreated GF WT mice following FMT from SPF WT and *Scgn*^-/-^ donors and *Scgn*^-/-^ mice in the Light-Dark Box Test. (n = 10 mice per group)

(G) Representative western blots of tryptophan metabolism-related enzymes in the mPFC of butyrate-treated and untreated GF WT following FMT from SPF WT and *Scgn*^-/-^ donors and *Scgn*^-/-^ mice.

(H-I) Quantification of IDO1(H) and KYNU (I) protein expression levels in the mPFC. (n = 5 mice per group)

(J-K) Concentration of L-kynurenine (J) and quinolinic acid (K) in the mPFC of butyrate-treated and untreated GF WT following FMT from SPF WT and *Scgn*^-/-^ donors and *Scgn*^-/-^ mice(n = 5 mice per group)

Data are presented as box plots showing median and interquartile range; individual data points represent biological replicates.

Statistical analysis:

One-way analysis of variance (ANOVA) followed by Tukey’s post hoc test : C-F and H-K.

Fig. S11 *Odoribacter splanchnicus* (OD) reverses anxiety-like behavior and kynurenine pathway changes induced by SCGN-deficient mice.

(A) Blood glucose levels during glucose tolerance test in OD -treated and untreated SPF WT and *Scgn*^-/-^ mice. (n = 6 mice per group).

(B) Area under the curve (AUC) for blood glucose during the glucose tolerance test in OD -treated and untreated SPF WT and *Scgn*^-/-^ mice. (n = 6 mice per group).

**(C)** Representative movement trajectories of OD -treated and untreated SPF WT and *Scgn*^-/-^ mice during the Open Field Test are depicted.

**(D)** The total distance traveled by OD -treated and untreated SPF WT and *Scgn*^-/-^ mice in the Open Field Test is quantified (n = 10 mice per group).

**(E)** The duration spent in the center area during the Open Field Test by OD -treated and untreated SPF WT and *Scgn*^-/-^ mice is reported (n = 10 mice per group).

**(F)** The duration spent in the open arms of the Elevated Plus Maze Test by OD -treated and untreated SPF WT and *Scgn*^-/-^ mice is quantitatively assessed (n = 10 mice per group).

**(G)** The counts of compartment transitions observed by OD-treated and untreated SPF WT and *Scgn*^-/-^ mice during the Light-Dark Box Test are reported (n = 10 mice per group).

**(H)**The concentrations of butyrate in feces from SPF WT, *Scgn*^-/-^ and OD -treated *Scgn*^-/-^ mice are reported. (n = 4 mice per group)

**(I)** The concentrations of butyrate in plasma from SPF WT, *Scgn*^-/-^ and OD -treated *Scgn*^-/-^ mice are reported. (n = 4 mice per group)

Data are presented as box plots showing median and interquartile range; individual data points represent biological replicates. NS, not significant.

Statistical analysis:

One-way analysis of variance (ANOVA) followed by Tukey’s post hoc test :B,and D-I.

Fig. S12 Effects of butyrate on pyramidal neuron firing and anxiety-related interneuron numbers across groups

**(A)** Heatmaps of normalized firing rates for interneurons in butyrate-treated and untreated SPF WT and *Scgn*^-/-^ mice during the 3-second window preceding and following transitions into closed and open arms across different groups ( n = 18-23 cells from 10 mice per group)

**(B)** Quantification of anxiety-related interneurons in butyrate-treated and untreated SPF WT and *Scgn*^-/-^ mice. (n = 18-23 cells from 10 mice per group)

**(C)** Representative traces of spontaneous inhibitory postsynaptic currents (sIPSCs) in butyrate-treated and untreated SPF WT and *Scgn*^-/-^ mice. Scale bars: 500 ms (horizontal), 20 pA (vertical).

**(D)** Cumulative distribution of sIPSC amplitudes recorded from pyramidal neurons in both butyrate-treated and untreated SPF WT and *Scgn*^-/-^ mice ( n = 15 cells from 5 mice per group).

**(E)** Mean sIPSC amplitudes observed in pyramidal neurons from butyrate-treated and untreated SPF WT and *Scgn*^-/-^ mice (n = 15 cells from 5 mice per group).

**(F)** Cumulative distribution of inter-event intervals of sIPSCs in pyramidal neurons from both butyrate-treated and untreated SPF WT and *Scgn*^-/-^ mice. (n = 15 cells from 5 mice per group).

**(G)** Mean sIPSC frequencies measured in pyramidal neurons from butyrate-treated and untreated SPF WT and *Scgn*^-/-^ mice. (n = 15 cells from 5 mice per group).

Data are expressed as mean ± SEM, with individual data points representing biological replicates. NS, not significant.

Statistical analysis:

One-way analysis of variance followed by Tukey’s post hoc test was conducted for panels B and D-G. Fisher’s exact test: F.
